# Structural Basis for the Recognition of Cellular mRNA Export Factor REF by Herpes Viral Proteins HSV-1 ICP27 and HVS ORF57

**DOI:** 10.1371/journal.ppat.1001244

**Published:** 2011-01-06

**Authors:** Richard B. Tunnicliffe, Guillaume M. Hautbergue, Priti Kalra, Brian R. Jackson, Adrian Whitehouse, Stuart A. Wilson, Alexander P. Golovanov

**Affiliations:** 1 Faculty of Life Sciences and Manchester Interdisciplinary Biocentre, University of Manchester, Manchester, United Kingdom; 2 Department of Molecular Biology and Biotechnology, University of Sheffield, Sheffield, United Kingdom; 3 Institute of Molecular and Cellular Biology, Faculty of Biological Sciences and Astbury Centre for Structural Molecular Biology, University of Leeds, Leeds, United Kingdom; University of North Carolina at Chapel Hill, United States of America

## Abstract

The herpesvirus proteins HSV-1 ICP27 and HVS ORF57 promote viral mRNA export by utilizing the cellular mRNA export machinery. This function is triggered by binding to proteins of the transcription-export (TREX) complex, in particular to REF/Aly which directs viral mRNA to the TAP/NFX1 pathway and, subsequently, to the nuclear pore for export to the cytoplasm. Here we have determined the structure of the REF-ICP27 interaction interface at atomic-resolution and provided a detailed comparison of the binding interfaces between ICP27, ORF57 and REF using solution-state NMR. Despite the absence of any obvious sequence similarity, both viral proteins bind on the same site of the folded RRM domain of REF, via short but specific recognition sites. The regions of ICP27 and ORF57 involved in binding by REF have been mapped as residues 104–112 and 103–120, respectively. We have identified the pattern of residues critical for REF/Aly recognition, common to both ICP27 and ORF57. The importance of the key amino acid residues within these binding sites was confirmed by site-directed mutagenesis. The functional significance of the ORF57-REF/Aly interaction was also probed using an *ex vivo* cytoplasmic viral mRNA accumulation assay and this revealed that mutants that reduce the protein-protein interaction dramatically decrease the ability of ORF57 to mediate the nuclear export of intronless viral mRNA. Together these data precisely map amino acid residues responsible for the direct interactions between viral adaptors and cellular REF/Aly and provide the first molecular details of how herpes viruses access the cellular mRNA export pathway.

## Author Summary

When invading host cells, herpes viruses highjack cellular components to allow them to replicate. It has been long recognized that each herpes virus has a specific signature adaptor protein which, among other functions, inserts viral mRNA into the cellular mRNA nuclear export pathway, enabling production of viral proteins by the host cell. This process has been extensively studied *in vivo* and *in vitro*, but despite many efforts, the molecular and structural mechanisms of key interactions between viral adaptors and cellular mRNA export factors have not been described. Here we present the first atomic-resolution structure of the key complex between the archetypal viral adaptor ICP27 (from Herpes simplex virus 1) and the cellular mRNA export factor REF, responsible for introducing viral mRNA into the cellular nuclear export pathway. We demonstrate that despite the absence of obvious sequence similarity, the adaptor protein ORF57 from a different herpes virus (Herpesvirus saimiri) binds REF in the same site and in a similar way. We have identified and studied amino acid residues responsible for REF recognition. Together the data provide the first molecular insight into how herpesviral signature proteins recognize cellular proteins, obtaining access to the cellular mRNA export machinery.

## Introduction

All herpesviruses replicate in the host cell nucleus and therefore utilise the host cell's protein transcription and translation apparatus, while at the same time suppressing the correspondent cellular processes [1]–[5]. Crucially, non-spliced viral mRNA is directed into the cellular mRNA export machinery, thus bypassing the stringent cellular controls which normally ensure that only fully processed mRNA is exported from the nucleus to the cytoplasm. In an uninfected cell, the process of mRNA export is closely connected with mRNA processing and splicing, which in turn are coupled with transcription. Cellular mRNA export involves the assembly of a multi-protein transcription and export (TREX) complex containing the RNA export factor REF/Aly; this signals that processing is complete and the cellular mRNA is ready to be exported via a TAP/NXF1-mediated interaction with the nuclear pore [6]–[8]. TAP forms a heterodimer with p15 and binds nucleoporins via central and C-terminal UBA-like domains [9]. REF/Aly provides a crucial link between mRNA and TAP: the binding of mRNA and TAP to REF are mutually-exclusive. TAP binding to REF-mRNA complex triggers transfer of RNA from REF to TAP. While REF is bound, it switches TAP into a high-affinity binding mode for RNA [10]. Once the ribonucleoprotein complex reaches the nuclear pore, REF dissociates and the mRNA is transported to the cytoplasm [11]. It is also possible that other cellular mRNA export factors may fulfil a role similar to that of REF/Aly [12]–[14]. Unlike cellular mRNA, the herpesvirus mRNA is often unspliced, therefore it cannot acquire export marker proteins using the normal pathway, via coupled transcription, splicing and export. To facilitate the efficient export of intronless viral mRNA all herpesviridiae produce a multi-functional adaptor protein [15] that shuttles between the nucleus and cytoplasm [3], [4], [16], and bridges between the viral mRNA and components of the TREX complex such as REF/Aly, thus marking viral mRNAs for export via TAP/NFX1 [17], [18]. In Herpes Simplex Virus type I (HSV-1) the infected cell protein 27 (ICP27) acts as the adaptor [3], [19]. In Herpesvirus Saimiri (HVS), the prototype γ-2 herpesvirus with close similarity to human Kaposi's Sarcoma-associated herpesvirus (KSHV), this role is carried out by the ORF57 protein [4], [20]–[22].

The regions of ICP27 and ORF57 involved in REF binding have been studied by analysing the effects of polypeptide truncations. For ICP27 it has been inferred as amino acids (aa) 104–138 [19], [23]. Recent *in vivo* studies suggested that the RGG box aa 138–152, which is involved in viral mRNA binding [2], [24]–[26], is also involved in REF/Aly interactions [27]. However, earlier *in vitro* data indicate that the RGG region does not bind REF directly [19]. In ORF57 the interactions with REF and with viral mRNA were localised within aa 8–120 [22], [28], [29]. Thus the identified regions of ICP27 and ORF57 apparently perform a similar function (REF/Aly and viral mRNA binding), however these regions lack any obvious sequence similarity which would highlight a common REF-binding motif. Moreover, it was not known whether ICP27 and ORF57 bind REF in a similar way. A number of previous studies used deletion mutants of REF to locate viral binding sites [22], [30], [31], however in the absence of structural information at that time, these mutations inadvertently perturbed the spatial structure of REF. The solution structures of murine Aly containing only the folded RRM domain [32] and the functional fragment of REF2-I which contained both the RRM and N-terminal domains (residues 1–155) have since been determined and characterised [33]. The REF2-I RRM domain at the surface-exposed area of α-helices 1 and 2 contains overlapping secondary binding sites for TAP and UAP56/DDX39; in the free form this binding site is shielded by loose binding of the N-terminal helix [33]. Additionally, this RRM has a non-canonical secondary RNA-binding site comprised of the loop regions [33]. The site of viral adaptor binding however remained unknown, making it difficult to understand how the assembly of the viral mRNA-protein complex is achieved.

Here we apply NMR spectroscopy to explore the binding of ICP27 and ORF57 with REF at a residue-level resolution and report a side-by-side comparison of the essential peptide fragments of ORF57 and ICP27 required for binding with REF. We demonstrate that the REF recognition site of ICP27 is very short but highly specific. The atomic resolution structure of REF RRM domain bound with the fragment of the viral protein adaptor is presented. The respective REF-binding site on ORF57 is longer and includes several weaker points of contact. The two viral proteins however bind at the same site on the REF RRM domain, which overlaps with the secondary TAP-binding site. The identified key residues of ORF57 for its interaction with REF are confirmed by side-directed mutagenesis and *ex vivo* studies.

## Results

### Initial identification of binding domains

**Figure 1:**
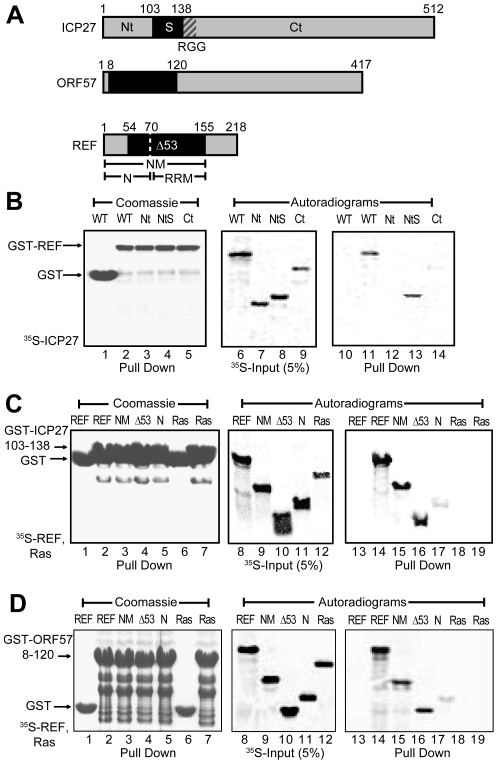
Identification of regions for the interactions between REF and ICP27/ORF57. (**A**) Subdivision of ICP27, ORF57 and REF2-I in fragments. (**B**) GST-REF pulls down ICP27^1–138^ (*NtS*) as efficiently as full-length ICP27, but not ICP27^1–103^ (*Nt*) and ICP27^139–512^ (*Ct*), indicating that residues 104–138 of ICP27 are involved in REF binding. (**C**) Fusion of the ICP27^103–138^ peptide to GST allows a specific pull down of REF, REF^1–155^ (*NM*) and REF^54–155^ (*Δ53*) and a very weak pull down of the REF^1–70^ (*N*); no interaction is detected with the control Ras protein. (**D**) Same pull down assay as panel **C** but using GST fusion of ORF57 aa 8–120 in place of ICP27.

To confirm the position of REF-binding domains within ICP27 and ORF57 and to minimise the size of constructs for more detailed NMR mapping, a series of fragments derived from HSV-1 ICP27 were screened for binding to GST-REF2-I using pull down assays (Fig. 1). The binding of ICP27 aa 1–138 (ICP27^1–138^) was essentially the same as that of the full-length protein, whereas ICP27^1–103^ and ICP27^139–512^ showed no binding (Fig. 1B), confirming aa 104–138 contain the REF interaction site *in vitro*, in agreement with previous studies [19], [23]. The GST fusion of ICP27^103–138^ was found to interact similarly with full-length REF^1–218^, REF^1–155^ and REF^54–155^ under the given conditions, but only weakly with REF^1–70^. These data indicate aa 104–138 of ICP27 are necessary and sufficient for interaction with REF (Fig. 1C). The REF-binding fragment of ORF57 aa 8–120 identified previously [22] similarly interacted with the same fragments of REF (Fig. 1D). Unlike an earlier study [22], the REF^54–155^ construct used here does not perturb the structure of the RRM domain [33]. These experiments showed that the main binding sites for both viral proteins are located within the REF^54–155^ construct.

To compare the mode of interaction of ICP27 and ORF57 with REF in more detail, NMR chemical shift mapping of backbone amides of ^15^N-labelled REF^1–155^ and REF^54–155^ was carried out (see Supporting Information available online, Fig. S1), by adding unlabelled ICP27^1–138^ or ORF57^8–120^ (Fig. S2) and monitoring signal shifts in ^1^H-^15^N correlation spectra. The sequence-specific signal assignment of REF in free form [34] was used to identify amino acids affected by binding. This indicated that while there may be a weak transient interaction with the N-terminal region of REF, especially for ORF57, the main interaction site is located in the REF RRM domain. The similar pattern of residues showing changes in chemical shifts induced by ICP27 and ORF57 indicated that both viral proteins bind REF on the same site (Supporting Information, Fig. S1E). No significant changes were observed in the ^1^H-^15^N correlation spectra of ^15^N-labelled REF^156–218^ upon addition of ICP27^1–138^ or ORF57^8–120^, confirming that the REF C-terminal domain is not involved in binding with these viral protein constructs (Fig. S1F). These initial studies thus confirm ICP27 and ORF57 bind REF in a similar manner.

### Detailed mapping of binding sites

**Figure 2:**
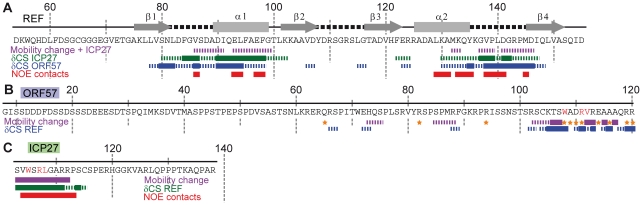
Identifying interaction sites within REF, ORF57 and ICP27 constructs via different NMR parameters. The detailed data is presented in SI. Purple solid and broken bars mark residues with large and moderate reduction of mobility upon binding, respectively, as evidenced by the increase in ^15^N{^1^H} NOE. Orange stars mark residues with amide signals broadened beyond detection in the complex. Large and moderate chemical shift changes (δCS) of amide signals for each ligand as labelled are shown as solid and broken bars, respectively. Red bars mark residues forming direct inter-molecular NOE contacts in REF-ICP27 complex. (**A**) Changes in REF^54–155^ induced by addition of ICP27^103–138^ and ORF57^8–120^. Position of secondary structure elements of REF (β-sheets, α-helices and loops) is shown in relation to its sequence. ICP27 and ORF57 bind on the same site on the REF RRM domain, with α-helices 1 and 2 and adjacent loop regions. (**B**) Changes in ORF57^8–120^ upon addition of REF^54–155^. The main REF-interaction site is mapped to residues 103–120. (**C**) Changes in ICP27^103–138^ induced by addition of REF^54–155^. The REF-binding site is mapped to residues 104-112.

**Figure 3:**
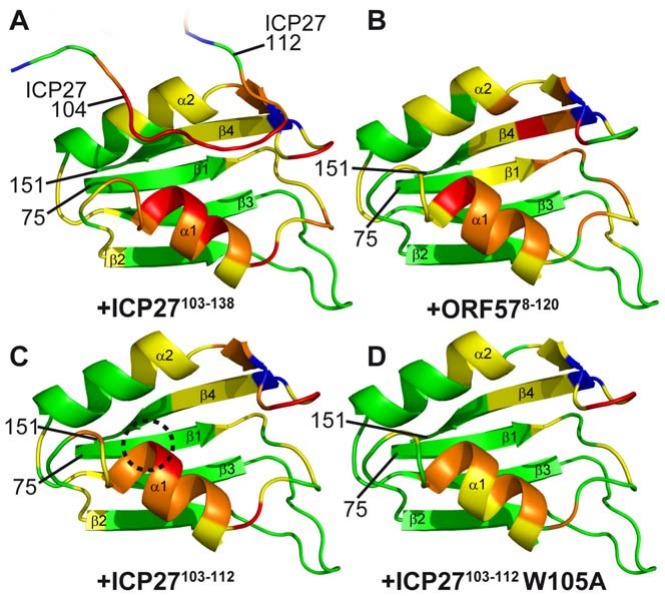
Mapping of the ICP27- and ORF57-induced signal shifts onto the structure of REF RRM domain. Backbone amide weighted chemical shift changes (δCS) are emphasized by colour. δCS >0.3 are red, 0.15–0.299 orange, 0.05–0.149 yellow, prolines are blue and regions unaffected are green. (**A**) Regions affected by addition of ICP27^103–138^. True ICP27 chain position determined based on NOE data is also shown for reference, with corresponded chemical shift changes due to REF binding similarly mapped. (**B**) Similar regions are affected by ORF57^8–120^ and (**C**) synthetic peptide ICP27^103–103^. (**D**) Synthetic mutant peptide ICP27^103–103^ W105A binding affects the same RRM regions, however the value of shifts at the top of α-helix 1 is reduced, highlighting the binding site for W105 (encircled on panel **C**). The position and numbering of N- and C- terminal residues are indicated.

To identify which amino acids of ICP27^103–138^ and ORF57^8–120^ bind REF^54–155^, the sequence-specific backbone assignment of free and bound forms of all these constructs was completed. Titrations were performed using additions of non-labelled polypeptides to ^15^N-labelled constructs, while monitoring spectral changes in ^1^H-^15^N correlation spectra. ICP27^103–138^/ORF57^8–120^ was added to REF^54–155^, and vice versa. This enabled the mapping of interaction sites on all proteins at a residue-level resolution. The values of heteronuclear ^15^N{^1^H} NOE were also measured to identify the parts of polypeptide chains with altered mobility due to binding (data overview on Fig. 2, with the more detailed data included in the Supporting Information). Titration of REF with ICP27^103–138^ confirmed that this short peptide interacted with REF in the same manner as the longer ICP27^1–138^ construct, and also in a similar manner as ORF57^8–120^. The viral protein binding site on REF RRM was mapped to α-helices 1 and 2 plus the adjacent loop regions (Fig. 2A and Fig. S3). The converse titration showed that only a short section of ICP27^103–138^, namely aa 104–112, displayed chemical shift changes and decreased mobility, whereas the rest of the peptide remained flexible in complex (Fig. 2C and Fig. S4). Similarly, addition of REF^54–155^ caused significant changes in signal positions and signal broadening, along with decreased mobility, primarily within a short section of residues 103–120 of ORF57^8–120^ (Fig. 2B and Fig. S5), the rest of the peptide was only weakly affected by binding. The REF-binding site of ORF57 thus appears to be significantly longer than that of ICP27, however both viral peptides bind to the same site on REF (Figs. 3A,B). Unlike the ICP27^103–138^ -REF^54–155^ complex, in the ORF57^8–120^ - REF^54–155^ complex a number of signals are broadened beyond detection, indicating that the latter complex is in the intermediate chemical exchange regime and hence is not suitable for atomic-resolution structural studies.

### Structure of the REF-ICP27 complex

**Figure 4:**
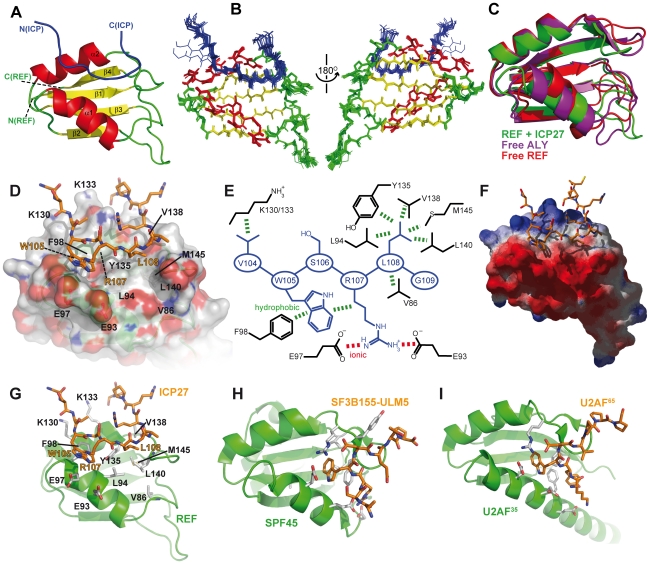
Structure of the REF - ICP27 complex. (**A**) Ribbon representation showing ICP27 coloured blue, and REF RRM coloured green, red and yellow for looped, α-helical and β-sheet regions, respectively. Positions of N- and C-termini of polypeptide chains are labelled. (**B**) Overlay of 20 lowest energy structures with backbone shown in the same orientation, and rotated 180°. The best-fit superposition is made using heavy backbone atoms of structurally defined regions aa 74–152 of REF and 102–112 of ICP27. Colour-coding is the same as on panel **A**. (**C**) Overlay of the RRM domains of free REF2-I (red, PDB code 2F3J), free murine Aly (purple, PDB code 1NO8) and ICP27-bound REF2-I determined here (green), demonstrating the shift in α-helix 1 position. (**D**) Representation of REF – ICP27 complex in the same orientation with partially transparent surface. (**E**) Schematic of the REF and ICP27 binding site. ICP27 residues are coloured blue and REF in black; the hydrophobic and electrostatic interactions are indicated by dashes coloured green and red, respectively. (**F**) Electrostatic surface with negative and positive charge coloured red and blue, respectively. Bottom row shows for comparison the known structures of RRM domains (green) with bound peptide ligands (orange). (**G**) REF^54–155^ in complex with ICP27^103–138^, determined here. (**H**) UHM domain of human SPF45 in complex with SF3B155-ULM5 (PDB code 2peh, [44]). (**I**) U2AF^35^ in complex with U2AF^65^ (PDB code 1jmt, [42]).

To obtain a detailed view of the ICP27^103–138^ interaction with REF^54–155^ we determined the atomic-resolution structure of this complex (Fig. 4A–G). The structure of the complex is well-defined owing to a large number of intra- and inter-molecular NOEs observed and assigned (Table 1; also Fig. S6). In agreement with the chemical shift mapping data, the viral peptide binds as a linear chain along the cleft formed by two α-helices on the surface of the RRM domain, which largely preserves its structure. However in the bound state the α-helix 1 of REF is shifted by approximately 3 Å (Fig. 4C). This shift causes some rearrangements within the looped regions, especially aa 136–146. These changes are accompanied by a noticeable decrease in mobility within the residues 86–90, 93–99, 132 and 137–145 as evidenced by ^15^N{^1^H} NOE data (Fig 2A). The shift in α-helix 1 exposes the sidechain of F98 for interaction with W105 of the peptide, and brings closer the sidechains of a hydrophobic patch composed of L94, Y135, V138, L140 and M145 (Fig. 4D, E). The upper edge of α-helix 1 contains a negatively charged patch of D90, E93 and E97 (Fig. 4F). The extended region aa 104–109 of ICP27 sitting along the REF cleft is followed by a loose bend at 109–112 which makes additional contacts with REF and points the remainder of the peptide chain away from the site. The region aa 114–138 remains flexible and hence does not participate in binding, in agreement with a lack of intermolecular NOEs and absence of signal shifts. From a structural perspective, three ICP27 residues appear most important for the interaction, forming a recognition triad (Fig. 4E). The sidechain of W105 makes hydrophobic contacts with F98 of REF and also with the aliphatic part of the R107 sidechain of ICP27. R107 forms salt bridges with the acidic residues of REF α-helix 1 (E93 and/or E97, the latter is the most likely binding partner in light of the decrease in mobility observed). L108 fits into a hydrophobic pocket composed of REF residues V86, L94, Y135, V138, L140, M145. Additionally V104 may also play a role in stabilising the complex, by forming hydrophobic contacts with the aliphatic parts of K130 and K133 of REF. The sidechain of S106 is solvent exposed and does not appear to directly bind REF. The ICP27 binding site shows a surprising degree of complementarity to REF, with a very short sequence used for highly specific recognition.

**Table 1:** NMR calculation statistics for an ensemble of the 20 lowest energy structures of REF^54–155^ bound to ICP27^103–138^.

Total number of NMR restraints	2500
Number of NOE restraints	2358
Intra-residue	558
Sequential	713
Medium range (2≤i≤4)	367
Long range intramolecular (5≤i)	622
Intermolecular	98
Dihedral	108
Hydrogen bonds	34
Mean number of NOE violations >0.1 Å	0.0189±0.0009
Mean number of dihedral violations >5°	0.7996±0.1434
Mean Cyana target function, Å^2^	6.20±0.38
Coordinate precision, Å	
RMSD (REF^74–152^ + ICP27^102–112^)	
Backbone	0.25±0.08
Heavy atom	0.79±0.11
RMSD (REF^74–152^)	
Backbone	0.21±0.07
Heavy atom	0.72±0.08
RMSD (ICP27^102–112^)	
Backbone	0.55±0.18
Heavy atom	1.32±0.31
Ramachandran plot (REF^74–152^ + ICP27^102-112^), % residues in regions:	
Favoured	77.5
Additional	22.2
Generous	0.3
Disallowed	0.0

### Molecular modelling of human Aly-ICP27 complex

**Figure 5:**
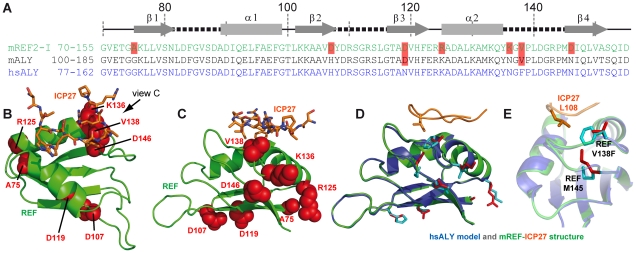
Modelling of complex between human Aly and HSV-ICP27. (A) Sequence alignment of RRM domains of murine REF2-I (mREF2-I: CAB76384) used in this study, murine Aly (mALY: AAC53117) and human Aly (hALY: AAD09608). Position of secondary structure elements (β-sheets, α-helices and loops) is shown in relation to the REF2-I. Resides in mALY and mREF2-I that differ from hALY are highlighted in light red. (B) A cartoon of the structure of REF-ICP27 with the position of the 7 amino acid differences indicated using red space-fill spheres, this orientation shown is the same as used in Fig. 3, whereas in (C) an alternative orientation is used for clarity. Only one amino acid difference is part of the ICP27 interaction site, namely Val138 of mREF2-I, which is a Phe in hALY. (D) A model of hsALY (blue) overlaid with the experimental structure of mREF2-I (green), with ICP27 also shown (orange). Residues that differ between the RRM domains of mREF2-I and hsALY are red sticks in the mREF2-I form and cyan sticks in hsALY. The sidechain of M145 altered in the modelling procedure is indicated by sticks. Also the ICP27 sidechain of L108 is indicated (orange) which is positioned within the hydrophobic pocket of REF. (E) Detailed view of the sidechains of Met 145, and Val138 and Phe138 in the modelled hsALY (blue) and experimental REF2-I (green) structures, plus L108 of ICP27 is shown (orange).

Herpes simplex virus (HSV) causes common infections in humans that occur on the mouth and lips, including cold sores and fever blisters. Although murine REF2-I protein employed in this study is commonly used as a model to study mRNA export, potentially there may be differences in the way ICP27 recognises its native partner Aly, the human orthologue of murine REF. Here we explored this issue in detail. A sequence alignment of murine REF2-I, murine Aly (mAly) and human Aly (hsAly) (Fig. 5A) show very high level of homology within the RRM domains. Specifically there are seven amino acid substitutions between murine REF2-I and human Aly (Fig. 5A). However, only one of these substitutions lies within a binding site (Fig. 5B and C), namely V138 (which is a phenylalanine in human Aly). This conservative substitution is positioned on the periphery of the hydrophobic pocket that contacts L108 of ICP27. Molecular modelling of the structure of human Aly bound to ICP27 was performed to see how significantly the binding interface with ICP27 is affected by the differences in sequence (Fig. 5D and E). The modelling results show that the increase in hydrophobic sidechain volume of the V138F mutation could be readily accommodated by the movement of the sidechain of M145 (Fig. 5E). All other amino acid substitutions were positioned away from the binding interface. Comparison of modelled hsAly-ICP27 and experimental murine REF-ICP27 solution structure showed a heavy atom backbone RMSD of only 0.04 Å, with the architecture of ICP27 binding site maintained in both homologues. Therefore we conclude that ICP27 can bind to human Aly in the same manner as to murine REF2-I.

### Exploring the specificity of REF binding site of viral protein adaptors using synthetic peptides

The characteristic triad, Trp followed shortly by Arg and then by a hydrophobic residue, is also found in the REF-binding region of ORF57, and bears distant similarity to the sequences of some other viral protein adaptors (Table 2 and Fig. S2). To probe the specificity of recognition, 12 synthetic peptides were tested for binding with REF^54–155^ (see Table 2). The first set of peptides was derived from ICP27 and included WT ICP27^103–112^ and its three single point mutants W105A, R107A and L108A, plus a shorter WT ICP27^103–110^ peptide with two arginines removed. The second set was derived from HVS ORF57 and included WT HVS-ORF57^103–120^, and its W108A, R111A and V112A mutants, along with a shortened peptide WT HVS-ORF57^105–115^ designed to probe the minimal binding region of ORF57 for REF. Additionally, two peptides were chosen from other viral proteins with apparent sequence similarity but containing some variations in the triad residues, to test how well these can bind REF in our experiments. We used sequences from Varicella-zoster virus (VZV) ORF4^108–119^ (Tyr instead of Trp) and Kaposi's sarcoma-associated herpesvirus (KSHV) ORF57^100–110^ (Tyr instead of Trp, and Lys instead of Arg). No prior data was available whether this VZV-ORF4 fragment binds to the RRM domain of REF. For KSHV-ORF57 a different region was previously implicated in binding with REF [35], [36], therefore the peptide KSHV-ORF57^100–110^ was not expected to interact and was used here as a negative control.

**Table 2:** Dissociation constants for the interaction of REF^54–155^ with viral protein fragments.

Peptide	*K*_D_ (µM)	Peptide sequence
ICP27^103–138^	28±8.7	GPLGSV**W**S-**RL**GARRPSCS…
ICP27^103–138^ S114E	17±7.9	GPLGSV**W**S-**RL**GARRPECS…
ICP27^103–110^	25±7.0	SV**W**S-**RL**GA
ICP27^103–112^	99±30	SV**W**S-**RL**GARR
ICP27^103–112^ W105A	550±210	SV**A**S-**RL**GARR
ICP27^103–112^ R107A	>10000^a^	SV**W**S-**AL**GARR
ICP27^103–112^ L108A	>5000^a^	SV**W**S-**RA**GARR
HVS ORF57^8–120^	78±12	…SCKTS**W**AD**RV**REAAAQRR
HVS ORF57^103–120^	45±15	SCKTS**W**AD**RV**REAAAQRR
HVS ORF57^103–120^ W108A	>7500^a^	SCKTS**A**AD**RV**REAAAQRR
HVS ORF57^103–120^ R111A	254±50	SCKTS**W**AD**AV**REAAAQRR
HVS ORF57^103–120^ V112A	156±32	SCKTS**W**AD**RA**REAAAQRR
HVS ORF57^105–115^	>1000^a^	KTS**W**AD**RV**REA
VZV ORF4^108–119^	>1000^a^	TG**Y**A-**RI**ERGHRR
KSHV ORF57^100–110^	>100000^a^	NR**Y**GK**KI**KFGT

Sequences of synthetic and recombinant polypeptides tested here are shown aligned according to position of triad residues (highlighted bold). Continuation of amino acid sequence in the construct is indicated as ellipsis. Mutated residues are underlined.

a Estimate of *K*_D_ obtained without curve fitting.

A separate titration of each peptide was carried out under the same sample conditions. Increasing amounts of peptide were added to a ^15^N-REF^54–155^ sample, achieving binding saturation whenever possible. Throughout these titrations amide chemical shift changes were monitored to assess the relative binding affinity of these peptides for REF and simultaneously map their binding sites. Estimates of dissociation constants were obtained for each peptide, and for the longer viral adaptor fragments (Table 2). The WT ICP27 peptides 103–112 and 103–110 showed very similar binding characteristics to the longer aa103-138 construct, confirming that this peptide comprises the entire binding site (Fig. S3). The mutant peptide W105A showed reduced affinity to REF^54–155^ but still bound with similar chemical shift change pattern (Fig. 3D and Fig. S3). A reduction in affinity was more pronounced in the L108A mutant, with the R107A mutation virtually abolishing the binding. For the HVS peptides, ORF57^103–120^ bound with affinity comparable to ORF57^8–120^, whereas affinity was decreased approximately two orders of magnitude in the shortened fragment WT-ORF57^105–115^. This agrees with the NMR mapping data that a longer sequence from ORF57 (residues 103–120) is involved in REF binding. The ORF57 mutant peptides R111A and V112A showed significantly reduced affinity for REF^54–155^, whereas the W108A mutant showed virtually no interaction. The VZV-ORF4^108–119^ peptide bound only weakly to REF^54–155^, whereas the KSHV-ORF57^100–110^ peptide used here as a negative control did not bind noticeably to REF (Table 2). These latter two viral adaptors lack the signature Trp residue, and additionally in KSHV-ORF57 the Arg within the triad is replaced by Lys. As a further control, we also checked if binding of the viral peptides is specific to the RRM domain of REF, or if it can occur with RRMs of other proteins as well. The proteins SF2 [37] and 9G8 [38] bind TAP/p15 and have roles in splicing and mRNA export somewhat similar to that of REF/Aly. They also contain an RRM domain and are therefore structurally homologous to REF/Aly. To test if the same ICP27 motif could interact with these RRM domains, we added a 5-fold excess of WT ICP27^103–112^ peptide to ^15^N-labelled SF2 [37] and 9G8 [38]. However no significant spectral changes and hence no binding was observed (Fig. S1). These experiments confirmed that the recognition of the REF RRM domain by viral adaptor proteins is highly specific, and the isolated peptides ICP27^103–112^, ICP27^103–110^ and ORF57^103–120^ are able to bind REF as efficiently as longer fragments of these viral proteins.

### Probing the effect of phosphorylation of ICP27 using an S114E mutant

Recently it had been suggested that phosphorylation of S114 of ICP27 [39] may affect its interaction with REF. In the structure obtained here, this Ser is situated right on the edge of the binding interface. In order to probe the possible effect of its phosphorylation on the interaction with REF, the mutant ICP27^103–138^ S114E was produced to mimic the presence of the negative charge on the sidechain. Titration of ^15^N-labelled REF^54–155^ with unlabelled ICP27^103–138^ S114E revealed binding to the same site on REF (Fig. S3) and *K*_D_ estimation showed that the affinity was only marginally different from the wild type ICP27^103–138^ construct (Table 2). To determine if the S114E mutation had any effect on the structure of the ICP27 construct used, we assigned and compared the fingerprint ^1^H-^15^N correlation spectra of ^15^N-labelled ICP27^103–138^ S114E mutant with that of the WT. The spectra overlaid well for all residues apart from residue 114 itself and its immediate sequential neighbours. According to ^15^N{^1^H} NOE measurement, both peptides were flexible in the free form, hence no structural changes were detected due to mutation. Titration with unlabeled REF^54–155^ indicated that the signals from the same region (aa 104–112) as WT ICP27 are most perturbed, with only a relatively small signal shift observed for E114 itself. These data suggest that there are no significant changes in direct binding of ICP27 to REF RRM in the mutant which mimics phosphorylation of S114.

### Mutations of ORF57 and ICP27 residues identified by NMR affect binding of human Aly

**Figure 6:**
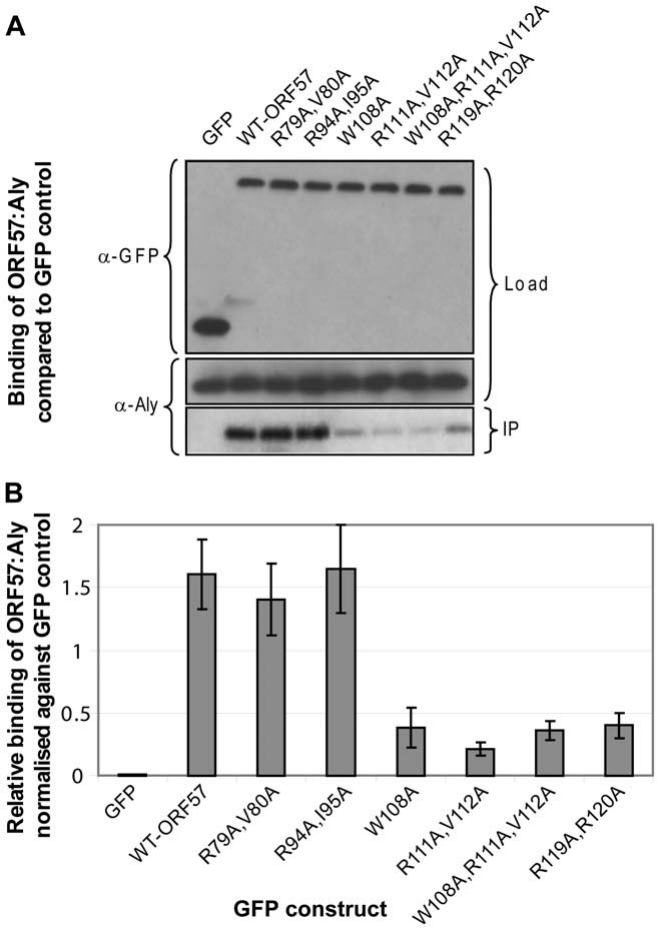
Functional importance of the identified REF binding site for the interaction of ORF57 with human Aly. (A) Co-immunoprecipitation assays were performed to compare the binding of wild type ORF57-GFP and ORF57 mutants. Site-directed mutations were made within the REF binding site of ORF57 and within its vicinity using Quik-Change II (Stratagene), according to the manufacturer's instructions. 293T cells were transfected with GFP-tagged ORF57 and mutant proteins and after 24 hours cell lysates were incubated with Protein A agarose and a polyclonal GFP-specific antibody and precipitated proteins were analysed by Western blotting with Aly-specific antibody and monoclonal antibodies to GFP as a loading control. (B) The relative binding affinities in repeated experiments were analysed quantitatively by densitometry. Point mutations affecting recognition triad residues 108, 111 and 112 and double arginines 119,120 all significantly reduce ORF57-Aly interaction. Mutations of similar type outside the interaction site have not affected the interaction between the two proteins.

To confirm the functional significance of critical residues within the REF binding site identified in ORF57 by chemical shift mapping experiments and analysis of synthetic peptide binding, a series of co-immunoprecipitation experiments were carried out using wild type and mutant forms of GFP-tagged full-length ORF57 and endogenous Aly in human cells (Fig. 6). Mutants were chosen that target the candidates for the recognition triad, as well as selected residues in the binding site and within the vicinity. All tested mutations within the proposed main binding site caused a significant decrease in ORF57-Aly affinity (namely, W108A, double R111A+V112A and R119A+R120A, and also triple W108A+R111A+V112A mutations, Fig. 6). The double mutants R79A+V80A and R94A+I95A situated outside the main binding site caused only a marginal if any decrease in Aly binding. These data corroborate the chemical shift mapping results and analysis of binding of synthetic peptide mutants, indicating that the main REF/Aly interaction site on ORF57 encompass aa 103–120, and confirms that triad residues W108, R111 and V112 of ORF57, in addition to R119 and R120, are important for the recognition of REF/Aly within the context of the functional full-length protein.

**Figure 7:**
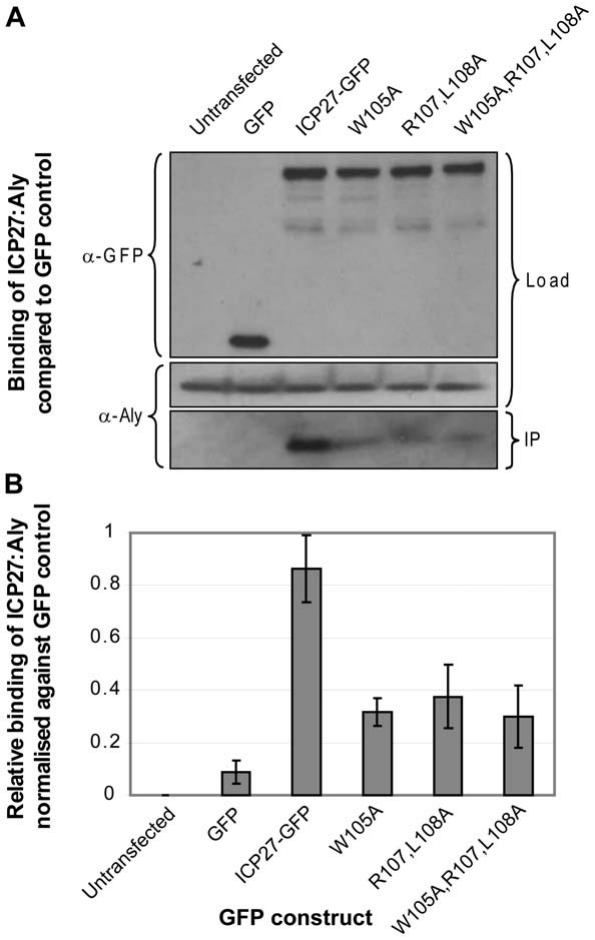
Functional importance of triad residues 105, 107 and 108 for the interaction of ICP27 with human Aly. (A) Co-immunoprecipitation assays were performed on 293T cells transfected with GFP-tagged ICP27 and its mutants. Mutations probed triad residues identified in the experimental structure formed between short protein fragments of ICP27 and REF. Cell lysates were incubated with Protein A agarose and a polyclonal GFP-specific antibody and precipitated proteins were analyzed by Western blotting with Aly-specific antibody and a monoclonal antibody specific to GFP as a loading control. (B) The relative binding affinities from 3 independent experiments were analysed quantitatively by densitometry. Point mutations of the REF recognition triad residues all significantly decrease binding between full-length ICP27 and endogenous Aly, confirming the functional significance of these residues identified by NMR.

Similar co-immunoprecipitation experiments were performed using wild type and mutant forms of full-length ICP27, specifically mutating W105A, R107A+L108A and W105A+R107A+L108A. Results demonstrate that all three mutants showed a significant reduction in Aly binding, again corroborating data obtained by chemical shift mapping and analysis of binding of synthetic peptide mutants (Fig. 7). The co-immunoprecipitation experiments for both ORF57 and ICP27 confirm that the REF-binding sites characterized here in detail using shorter polypeptide constructs are also functionally important for the interaction of these proteins with Aly/REF in their full-length native forms.

### Mutations of HVS ORF57 residues important for REF/Aly binding affect ORF57-mediated cytoplasmic accumulation of mRNA

**Figure 8:**
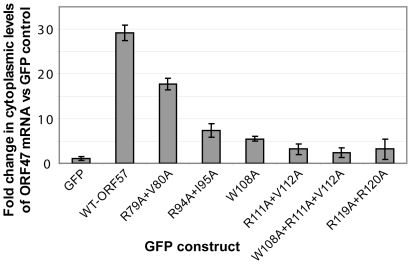
Perturbing the ORF57-REF/Aly interaction with point mutations decreases cytoplasmic accumulation of viral mRNA. 293T cells were transfected with the pORF47 reporter mRNA construct and the vectors containing wild type ORF57 or its mutants, and RNA was isolated from cytoplasmic fractions. The mutations probed residues from REF binding site, as well as from its vicinity. qRT-PCR was performed and data for mRNA reporter plus ORF57-transfected cells normalised against cells transfected with reporter in the presence of GFP. A ΔΔcT method was applied to determine the relative levels of reporter mRNA between samples. Point mutations of ORF57 residues implicated in direct interaction with REF, and reducing its binding, all caused significant decrease in cytoplasmic accumulation of viral mRNA.

The functional importance of ORF57 residues within REF-binding site were also measured via an *ex vivo* assay for cytoplasmic accumulation of an HVS ORF47 reporter mRNA (Fig. 8), using wild type and mutant ORF57 proteins, as previously described [36], [40]. As such, the cytoplasmic accumulation detected in this assay reflects the ability of ORF57 to form an export competent ribonucleoprotein particle. Human 293T cells were transfected with pORF47 (a plasmid expressing the late intronless ORF47 mRNA) in the presence of wild type or mutant ORF57 proteins. After 24 hours RNA was extracted from cytoplasmic fractions and levels assessed by qRT-PCR. The mutation of residues directly implicated in the REF/Aly interaction, namely W108A, R111A+V112A and R119A+R120A, and also W108A+R111A+V112A, all substantially reduced the efficiency of the mRNA cytoplasmic accumulation. In addition, mutations of residues outside the primary REF-binding site were tested. Mutation R94A+I95A also similarly reduced cytoplasmic accumulation, whereas R79A+V80A caused only a marginal decrease. R94 is situated just outside the main REF-binding site and is part of the nuclear localization signal, and the observed effect can be possibly explained by its involvement in the interaction with viral mRNA and/or perturbed nuclear localization. The small effect of R79 substitution may be due to possible changes in mRNA binding. The results of these *ex vivo* experiments confirm the functional importance of individual residues identified by NMR for specific binding in the context of native Aly and full-length ORF57. Moreover, the results suggest that these individual residues critical for the HVS ORF57 – REF/Aly interaction are also required to enable efficient cytoplasmic accumulation of viral mRNA in our assay. This confirms the functional significance of ORF57 – REF/Aly interaction for ORF57-mediated nuclear export of viral intronless transcripts, leading to recruitment of other hTREX proteins [40] and TAP.

## Discussion

The use of NMR with short optimised constructs of REF, HSV-1 ICP27 and HVS ORF57 has allowed the precise determination of the residues important for the recognition of viral proteins by the cellular mRNA export factor REF. Despite the lack of obvious sequence similarity, both viral proteins bind on the same main site, along the cleft formed by the two α-helices in the RRM domain of REF. Our data shows that for ICP27 a short but highly specific amino acid sequence 103–110 is required and sufficient for REF-binding (with residues 105, 107 and 108 being critical). This region is immediately followed by a nuclear localization sequence (NLS) aa110-137 [41], without a significant overlap between the two. Within the ORF57 protein, the REF-interaction sequence is significantly longer and includes aa 103–120. The REF interaction sites of both ICP27 and ORF57 proteins contain a recognisable triad pattern, a Trp shortly followed by an Arg-Leu/Val pair, which proved to be essential for REF binding. Mutation of these critical triad residues both in ICP27 and in ORF57 significantly reduced binding with REF. The insertion of an additional residue within this triad (as in the case of ORF57) distorts the complementarity of the binding interface and likely necessitates the presence of additional compensating contacts (i.e., via R119 and R120) and hence a longer recognition site. This is supported by chemical shift mapping, effect of peptide truncation on *K*_d_ and a change in REF binding for the R119,120A double mutant. The critical REF recognition residues were first identified and characterized in detail using relatively short protein constructs, confirming high specificity of detected interactions. It was however important to show that these REF recognition sites also work in the full-length native proteins. Here we demonstrate that the mutations of residues from recognition triads significantly reduced binding between full-length viral ICP27 and ORF57 and human Aly in co-immunoprecipitation assays, confirming functional significance of detected binding sites for proteins in their native form in nearly physiological conditions, both for ORF57 and ICP27.

The REF recognition site on ICP27 involves residues 103 to 110 (possibly extended to 112) and in our experiments it is entirely sufficient for highly specific binding with REF *in vitro*. Based on the interpretation of *in vivo* experiments, recently it had been suggested that phosphorylation of S114 [39] or modifications within the RNA-binding RGG motif aa 138–152 [27] affect the ICP27 interaction with REF. In the structure presented here, S114 is positioned very close to the binding site, but not immobilised upon binding. In principle, one can envisage that phosphorylation of this residue can make an additional favourable Coulombic contact with K133 and/or K136 of REF, immobilizing phosphoserine and strengthening the complex further. We have checked this hypothesis here by using a S114E mutant as a phosphoserine mimic. Both WT-ICP27^103–138^ and ICP27^103–138^S114E interact with REF with similar *K*_D,_ the mutant interacts only marginally stronger (Table 2). This insignificant change in affinity observed in our experiments and the position at the periphery of the REF-binding motif suggests that it is unlikely that modifications of S114 can provide a direct stringent control of the REF-ICP27 interaction. This agrees with the observation that the S114A mutant still co-immunoprecipitates with REF/Aly [39]. Similarly, the RNA-binding RGG motif (aa 138–152) is positioned sequentially away from the specific REF-binding motif. Modifications in this RGG motif are unlikely to have a direct effect on ICP27-REF binding. The effects of ICP27 modifications outside the main 103–110 site on REF binding recently observed *in vivo* [27], [39] can be alternatively explained by trapping the mutated ICP27 in complexes upstream of the pathway, as suggested by [39]. Additionally, modifications within the RGG region of ICP27 may affect binding with RNA; this could indirectly affect ICP27-REF affinity if the RNA bridges the two proteins. Further experiments, which take into account cellular availability of modified ICP27 for interaction with REF/Aly and the bridging role of mRNA, are needed to reconcile the *in vivo* and *in vitro* data.

Here we have presented the first atomic-resolution structure of the complex between the fragment of archetype viral signature protein, ICP27, and the cellular export factor REF2-I. The ICP27 peptide binds on the α-helical side of the RRM domain, along the crevice between α-helices. The position of this peptide is defined by the presence of multiple unambiguous NOE contacts, which in particular pinpoint the position of the W105 and L108 of ICP27 (Fig. S6D and Fig. S7) and therefore align the peptide along the crevice. The corresponding 3D structure of human Aly bound to ICP27 is currently unknown, but within the ICP27 binding site the two proteins differ by just one amino acid residue in position 138, with Phe for Aly and Val for REF2-I. This site is situated on the periphery of the hydrophobic patch which interacts with L108 of ICP27 (Fig. 5). Comparative modelling of human Aly suggested the mutation could be easily accommodated without disrupting the interaction, therefore the complex between human Aly and ICP27 is likely to be structurally very similar to the one between REF2-I and ICP27 determined here. This modelling provides a molecular level insight into how the ICP27 protein from Herpes Simplex virus may interact with human Aly, to facilitate the nuclear export of herpesviral intronless mRNA, an essential prerequisite for virus replication.

The previous examples of peptides bound on the α-helical side of RRM-type domains differ from the structure described here. The U2AF homology motifs (UHM) have been shown to recognize a Trp residue which is preceded by a stretch of basic residues [42]–[44]. In the UHM-type of recognition, the signature Trp sidechain of the peptide is inserted into the hydrophobic pocket formed mainly by the looped regions, with the bound peptide running almost perpendicular to the crevice between the two α-helices (Fig. 4H,I). The characteristic Arg-X-Phe motif situated in the loop shortly after α-helix 2 is the defining signature of UHMs and is the key to Trp recognition [43]. The RRM of REF2-I clearly lacks this motif, and therefore does not belong to UHM class. Moreover, the similar hydrophobic pocket in the REF RRM is occupied by Leu108 of ICP27, and not by Trp (Fig. 4G–I). Interestingly, the presence of the Trp appears not to be as crucial as the other triad residues involved in ICP27 recognition, as its mutation reduces binding only one order of magnitude (Table 2). Residues more important for ICP27 binding are Arg107 and Leu108. Unlike in UHM recognition, in the REF - ICP27 complex the Trp makes contacts mainly with the top of α-helix 1, and middle part of α-helix 2. Both the abundant NOE contacts (Figs. S6 and S7) and relative perturbations caused by the W105A mutation (Fig. 3C,D), all consistently indicate that the mode of ICP27 binding with REF is different from peptide recognition by UHMs. Recently another apparently similar complex between PTB-RRM2 and Raver1 peptide has been described by NMR and modelling [45], where a crucial Leu-Leu pair of the LLGxxP motif is inserted in the binding pocket in the loops adjacent to α-helix 2. In this modelled complex the peptide also has a different orientation, compared with our structure based on direct NOE restraints, and interacting motifs have little similarity. Therefore, the structure presented here displays another, previously undescribed, mode of peptide-RRM recognition, adding to the previously recognized diversity of RRM-ligand interactions [46].

Previously, the position of viral mRNA binding sites on ORF57 has been loosely mapped to aa8-120 [22], [28], [29]. As the REF binding site aa103-120 is situated within the same fragment, it is not clear yet whether RNA and REF/Aly binding to ORF57 occurs concurrently or cooperatively. Our further studies are aimed at clarifying this. In the case of ICP27, the viral mRNA binding site is situated within the RGG region shortly following the REF-binding site. One can therefore anticipate that ICP27 brings and introduces the viral mRNA to REF/Aly, which can bind both ICP27 (via RRM domain) and viral mRNA (via N- and C-termini) simultaneously, ensuring a multi-contact interaction interface.

Here we demonstrated that point mutants in positions 108, 111, 112, 119 and 120 that reduce the ORF57 - REF/Aly interaction also dramatically decrease the ability of ORF57 to promote the nuclear export of intronless viral mRNA. Therefore these residues are functionally important for mRNA export, likely by directly mediating recruitment of REF/Aly. The ability of ORF57 and homologues to interact with export adapter proteins, such as REF/Aly, and possibly functional homologues such as UIF [13], is therefore likely to be essential for the formation of an export competent ribonucleoprotein particle. This in turn is essential for efficient viral mRNA nuclear export and subsequent virus replication, as we have previously demonstrated that recruitment of the complete hTREX complex to viral intronless mRNAs is essential for both HVS and KSHV lytic virus replication [29], [35]. Similarly, mutations of ICP27 residues in positions 105, 107 and 108 have also been shown here to decrease the interaction between full-length ICP27 and human Aly. Further experiments are needed to confirm the effect of mutations of recognition triad residues on the viral mRNA export mediated by ICP27. The functional role of the REF/Aly binding regions in ICP27 export to the cytoplasm have been studied previously by deletion of polypeptide fragments. Specifically, ICP27 deletions 64–108 (d2-3) and 109–138 (d3-4) were used and interpreted as mutants perturbing interaction with REF/Aly [17]. The current work suggests that in fact only the first of these two deletions affected the REF/Aly recognition triad. In the second d3-4 construct the main REF/Aly binding site was completely preserved, while the NLS was perturbed. This may explain why the d3-4 mutant maintained efficient export of ICP27 to the cytoplasm [17] – the interaction of this construct with REF/Aly was in reality possible. Moreover, the deletion constructs said to be lacking the REF/Aly binding site and used to demonstrate the absence of REF/Aly bridging between ICP27 and TAP/NXF1 [17], in fact, inadvertently preserved this site. In view of the detailed data presented here on the exact point mutations (residues 105, 107 and 108) which will perturb interactions with REF/Aly without affecting the NLS aa110-137, further functional studies may be warranted to reconsider the suggested diminished roles of the ICP27 - REF/Aly interaction in cytoplasmic export of ICP27, and of REF/Aly in mediating interactions with TAP/NXF1 [17]. Such studies however should consider the possibility that other adaptor proteins [12]–[14] may substitute the function of REF/Aly *in vivo* once the ICP27 – REF/Aly interaction is blocked, complicating the analysis. Additional experiments are also required to assess and map interaction of ICP27 with functional homologues of REF/Aly such as the recently identified UIF protein [13], to explore the role of alternative pathways. Regardless of how essential the REF-viral protein interaction appears from siRNA evidence [14], the recruitment of the ubiquitously-present cellular export factor Aly/REF to viral ICP27/ORF57 can be envisaged as a highly useful pathway linkage, increasing an overall efficiency of viral mRNA export, due to the ability of this export factor to remodel TAP triggering high affinity RNA - TAP interactions [10]. The main interaction site for TAP on REF is an N-terminal arginine rich motif; however, the REF RRM also contributes to TAP interactions [10], [33] and this secondary site overlaps with the site recognised by ICP27 and ORF57. Therefore TAP recruitment is likely to lead to remodelling of the viral ribonucleoprotein complex. Although the partial displacement of the viral adaptor fragment from the surface of RRM of REF upon TAP binding may be possible, the complete displacement of the viral proteins from the ribonucleoprotein complex seems unlikely since a ternary complex of REF - TAP and ORF57/ICP27 assembles *in vitro* [19], [22]. Further studies are required to establish how the viral mRNA export complex is remodelled during export and which proteins contact the viral mRNA directly at each point in the export pathway.

## Materials and Methods

### Protein expression and purification

Constructs REF^1–155^, REF^54–155^, ICP27^1–138^ and ORF57^8–120^, expressed in pET24b (Novagen) vector, were produced as described previously [33], with additional purification on a Superdex 75 (GE Healthcare) column (GF buffer: 20 mM phosphate, 150 mM NaCl, 50 mM L-Arg/L-Glu/β-mercaptoethanol and 10 mM EDTA, pH 6.2). Proteins SF2 and 9G8 were purified as described previously [37], [38]. ICP27^103–138^ WT and S114E peptides were expressed as GST-fusions in a pGEX-6P-1 plasmid, and cleaved by PreScission protease on GSH resin according to standard protocol (GE Healthcare). Eluted peptide was supplemented with 5 mM DTT and protease inhibitor cocktail (Roche), and exchanged into GF buffer using an Amicon pressure cell with 1 k MWCO membrane via a series of dilutions/concentrations. A Sephacryl S-100 HR (GE Healthcare) gel filtration column was used to purify the peptide further. Peptide was >95% pure according to tricine-SDS-PAGE.

### Pull down assays and co-immunoprecipitations

GST or GST protein fusions were first immobilised on 30 µl slurry glutathione-coated beads (GE Healthcare) before 8 µl ^35^S-radiolabelled proteins synthesised in rabbit reticulocytes (Promega) were added to the binding reactions in RB100 buffer (25 mM HEPES pH 7.5/100 mM KOAc/10 mM MgCl_2_/1 mM DTT/0.05% Triton X-100/10% glycerol) in presence of 10 µg/ml RNAse A. Washed and eluted protein complexes were resolved on 15% SDS-PAGE stained with Coomassie blue and analysed by PhosphoImage. To analyse the effect of HVS ORF57 and HSV-1 ICP27 point mutations on Aly/REF binding, co-immunoprecipitation were performed as previously described [47], [48]. Human 293T cells were transfected with wild type GFP-ORF57 or GFP-ICP27 and respective mutants, generated using the QuickChange II site-directed mutagenesis kit (Stratagene), using Lipofectamine 2000 (Invitrogen, Paisley, UK), as per the manufacturer's instructions. Briefly, after 24 hours, cell lysates were harvested, precleared with Protein A agarose for 1 hour at 4°C and then incubated with polyclonal GFP-specific antibody for 2 hours at 4°C. Protein A agarose was added to the cell lysates and incubated for a further 3 hours at 4°C. The agarose was washed 3 times to remove unbound protein. Western blot analysis was then performed using an Aly-specific antibody and GFP-specific monoclonal antibody as a loading control. Densitometry analysis was then performed on 3 independent experiments using the ImageJ software.

### Cytoplasmic mRNA accumulation assay

293T cells were transfected with ORF57 or the respective mutants in the presence of the pORF47 reporter mRNA as previously described [49]. Cytoplasmic ORF47 mRNA levels were then assessed by qRT-PCR as previously described [36]. Briefly, after 24 hours, cells were lysed in 200 µl of PBS 1% Triton-X 100 (v/v) containing 40 U of RNAse Out (Invitrogen), and cytoplasmic fractions isolated using Trizol (Invitrogen) as previously described [36]. Total RNA (1 µg) from each fraction was reverse transcribed using Superscript II (Invitrogen) and 10 ng of cDNA used as template in SensiMix*Plus* SYBR qRT-PCR reactions (Quantace). qPCR was performed using the Rotor-Gene Q 5plex HRM Platform (Qiagen), with a standard 3-step melt program (95°C melt for 30 sec, 60°C annealing for 15 secs, 72°C extension for 20 secs). Following confirmation that qPCR efficiency was comparative between ORF47 and the reference mRNA (GAPDH), quantitative analysis was performed using ΔΔcT analysis as previously described [36].

### NMR experiments

All experiments were carried out at 30°C on Bruker DRX600, DRX700 and Varian Inova 800 MHz spectrometers equipped with cryoprobes. The weighted chemical shift changes of amide signals *δCS* caused by complex formation were measured as 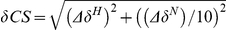, where Δδ^H^ and Δδ^N^ were changes in proton and nitrogen chemical shifts, respectively. Standard triple-resonance experiments were used to assign spectra of ICP27^103–138^, ORF57^8–120^ and REF^54–155^ in their free and bound states. Additionally, carbon-detection experiments (CON, CaCO, CbCaCO, CbCaCO(N), CbCaNCO) were used as an aid to the ORF57 assignment. Spectra were processed using NMRpipe [50] and Topspin 2.1 (Bruker) and analysed using Sparky (University of California). Distance restraints obtained from 3D ^15^N- and ^13^C edited NOESY-HSQC experiments (τ_m_ 120 ms) and dihedral restraints from TALOS [51] were used in structure calculations by CYANA [52]. Additionally, intermolecular contacts were unambiguously identified using ^13^C edited, ^12^C-filtered NOESY-HSQC (τ_m_ 150 ms) spectra acquired on Varian Inova 800 MHz spectrometer. In this experiment only NOE crosspeaks between ^1^H-^13^C moieties of ^13^C,^15^N-labelled REF and ^1^H(^12^C) of unlabelled ICP27 peptide were observed [53], [54]. A final ensemble contained 20 structures with lowest target function values. Images were generated using Pymol (DeLano Scientific). For the REF - ICP27 complex, structure coordinates and experimental constraints have been deposited into the Protein Data Bank and chemical shifts in the BioMagResBank (access numbers 2kt5 and bmr16683 respectively). Other chemical shift assignments deposited in the BioMagResBank are: free ICP27^103–138^ bmr16696, free REF^54–155^ bmr16697, and ORF57^8–120^ bmr16698 (both free and bound to REF^54–155^).

Unlabelled synthetic peptides were obtained from Peptide Protein Research Ltd (UK). The synthetic peptide sequences used were from Varicella-zoster virus ORF4^108–119^ (AAY57694), Kaposi's sarcoma-associated herpesvirus ORF57^100–110^ (YP_001129410), Herpesvirus Saimiri ORF57^103–120 & 105–115^ (CAC84353), Herpes simplex virus 1 ICP27^103–110 & 103–112^ (AAF43147). Dissociation constants (*K_D_*) were derived by monitoring chemical shift changes in ^1^H-^15^N correlation spectra of 100 µM ^15^N-REF^54–155^ as a function of increasing peptide concentrations, and fitting data to the standard equation [55]. For very weak binding peptides where chemical shift changes were too small to obtain a curve for fitting, a lower limit estimate of *K_D_* was obtained by comparisons of the magnitude of chemical shift change observed relative to those of stronger complexes.

### Molecular modeling

Comparative modelling of the human Aly – ICP27 complex was performed using Swiss-PdbViewer [56] and the lowest energy conformer of the REF - ICP27 complex as a template. Mutations A75G, D107H, D119N, R125K, K136N, V138F and D146N (which reflect the differences between murine REF2-I and human Aly within the RRM domain) were introduced. All mutations except V138F involved solvent exposed sites and did not cause steric clashes. For the V138F substitution, a conformation was chosen that minimised the number of steric clashes while orientating the aromatic sidechain towards the hydrophobic core of REF. An energy minimization was conducted to remove the remaining steric clash with the ε-methyl of M145, resulting in a change in the M145 side chain rotamer, and virtually no movement of the backbone (heavy atom backbone RMSD of 0.04 Å).

## Supporting Information

Figure S1 Overall identification of amino acid residues of REF2-I affected by binding with viral protein fragments plus SF2 and 9G8 spectra. Chemical shift changes within REF spectra were monitored upon addition of ICP27 or ORF57 constructs as an indication to which amino acids are involved in binding. Where the weighted chemical shift changes of amide signals *δCS* caused by complex formation were above 0.1, or the peak could not be followed due to broadening, an arrow is drawn. REF^1–155^ was titrated with ORF57^8–120^ (A) and ICP27^1–138^ (B), similarly REF^54–155^ was titrated with ORF57^8–120^ (C) and ICP27^1–138^ (D). Mapping of residues affected by binding is demonstrated (E). From the same titration data, ribbon representation of REF (i) is shown in the same orientation as surface representations showing charge (ii) with acidic and basic residues colored red and blue. Chemical shift changes from ORF57 (iv and vi) and ICP27 (iii and v) titrations are mapped with significant changes colored red. Labels used: NM, REF^1–155^; Δ53N, REF^54–155^. The similar pattern of shift changes throughout supports both ICP27 and ORF57 have the same main binging site situated in the folded RRM domain of REF. The changes to the chemical shifts within N-helix are likely to be caused by the release of N-helix which is normally bound to the same site in the free state of the protein [33]. (F) Overlay of ^15^N-HSQC spectra of the C-terminal region (residues 156–218) of REF2-I show no significantly changes from free form (red) upon addition of 2-fold excess of either ICP27^1–138^ (blue) or ORF57^8–120^ (green). Signals marked with asterisks originate from residues of C-terminal His-tag which are not part of REF. Additionally, ^15^N-HSQC spectra of the RRM domains of both 9G8 (G) and SF2 (H) show no changes upon addition of 2-fold excess of ICP27^103–112^ synthetic peptide.
(3.88 MB TIF)

Figure S2 Sequence alignment of N-terminal parts of ICP27 homologues from α and γ herpesviruses. The first 200 amino acids of HSV-1 ICP27 (AAF43147) containing the REF-interaction site was aligned manually with the predicted unstructured N-terminal regions of the proteins HSV-2 UL54 (NP_044525), EBV EB2 (YP_401659), HVS ORF57 (AAA46125), KSHV ORF57 (YP_001129410) and VZV ORF4 (NP_040127). REF-interacting regions identified in this study are shown in bold, and regions probed for REF-binding using synthetic peptides are underlined. There is very weak homology between export adaptors within the N-terminal regions shown, with the exception of the very closely related HSV-1 ICP27 and HSV-2 UL54 proteins.
(0.34 MB TIF)

Figure S3 More detailed analysis of REF^54–155^ interactions with viral protein fragments and their mutants using NMR spectroscopy. (A) Amino acid sequence of REF construct (shown in a zigzag fashion), with T7 and poly-His tags coloured blue. Secondary structure elements are indicated with helices as red blocks, sheets as yellow arrows and larger loops as dotted lines. (B) Chemical shift changes δCS in backbone amides of REF^54–155^ upon addition of peptides: ICP27^103–138^, green circles & dashed line; ORF57^8–120^, blue circles & dotted line; ICP27^1–138^, purple diagonal crosses; ICP27^103–112^ synthetic peptide, red squares; ICP27^103–112^ W105A synthetic peptide, yellow plus signs; ICP27^103–138^ S114E synthetic peptide, green triangles. All the peptides were added in a 5-fold molar excess with respect to REF. (C) Heteronuclear ^15^N [^1^H] NOEs measured for REF^54–155^ in absence (red circles) and presence (green triangles) of ICP27^103–138^ were used as a measure of mobility change upon binding. (D) Overlay of ^1^H-^15^N-correlation HSQC spectra of REF^54–155^ with increasing amounts of ICP27^103–138^ added. Signal assignment is shown. Spectra are coloured red though green for free to bound forms of REF, respectively. Spectra are shown for the ratios 1∶0, 1∶0.5, 1∶1, 1∶1.5 and 1∶2 (REF:ICP27). (E) Comparison of TROSY spectra of REF^54–155^ in free form (red), bound to ICP27^103–138^ (green), bound to ORF57^8–120^ (blue) suggests that the binding of ICP27 and ORF57 fragments affects essentially the same signals and hence occurs at the same binding site. All NMR experiments were carried out in the same NMR buffer (20 mM phosphate, 50 mM NaCl, 50 mM L-Arg/L-Glu/β-mercaptoethanol and 10 mM EDTA, pH 6.2 plus 10 mM DTT and 0.1% NaN_3_).
(1.79 MB TIF)

Figure S4 Analysis of ICP27^103–138^ interactions with REF^54–155^ using NMR spectroscopy. (A) Amino acid sequence of ICP27 construct. The sequence from the remaining PreScission protease cleavage site is coloured blue. (B) Chemical shift changes in backbone amides of ICP27^103–138^ upon addition of Ref^54–155^, red squares. The horizontal dashed lines represent thresholds for strong and medium shift changes used for creating the summary Fig. 2. (C) Heteronuclear ^15^N [^1^H] NOEs measured for ICP27^103–138^ in free form (green circles) and in presence of REF^54–155^ (red squares) were used to detect the change in polypeptide mobility. (D) Overlay of ^1^H-^15^N-correlation HSQC spectra of wild type ICP27^103–138^ (blue) with ICP27^103–138^S114E (red) in free form and ICP27^103–138^S114E with a 5-fold excess of REF^54–155^ added (green). The inset at the top right shows indole region of the spectrum. (E) Overlay of ^1^H-^15^N-correlation HSQC spectra of ICP27^103–138^ with various amounts of added REF^54–155^. Labels show sequence-specific signal assignment. Spectra are coloured blue (through green) to red for the free and complexed peptide respectively. For clarity only the 1∶0, 1∶0.25, 1∶0.5, 1∶0.75, 1∶2, and 1∶6 (ICP27:REF) spectra are shown. (F) Comparison of ^15^N-HSQC spectra of ICP27^103–138^ peptide in free (blue) and REF^54–155^ bound form (red) and ICP27^1–138^ in free (cyan) and REF^54–155^ bound form (orange). Assignments for bound form of ICP27^103–138^ are shown. The spectrum of ICP27^103–138^ overlays well with the spectrum of ICP27^1–138^, this suggests that the truncation does not disrupt the structure of the shorter construct. The REF-binding site of ICP27 ^1–138^ is all situated within the 103–138 fragment, as signals from other parts of this longer viral protein construct are not affected by binding with REF.
(1.42 MB TIF)

Figure S5 Analysis of ORF57^8–120^ interactions with REF^54–155^ using NMR spectroscopy. (A) Amino acid sequence of ORF57 construct, with T7 and poly-His tags coloured blue. (B) Chemical shift changes in backbone amides of ORF57^8–120^ upon addition of REF^54–155^ are shown as red triangles. The arrows identify residues broadened beyond detection in the bound state. The horizontal dashed lines represent thresholds for strong and medium shift changes used for creating the summary Fig. 2. (C) ^15^N [^1^H] NOEs measured for ORF57^8–120^ in free form (black crosses) and in presence of REF^54–155^ (red squares) identify regions with polypeptide mobility changed upon binding. Orange stars mark residues with amide signals broadened beyond detection in the complex, for these no NOE data could be obtained. (D) Overlay of ^15^N-HSQC spectra for titration of ORF57^8–120^ with REF^54–155^. Spectral assignment is shown. Spectra are coloured orange though blue for free to bound forms of REF respectively. Spectra are shown for the ratios 1∶0, 1∶0.15, 1∶0.3, 1∶1, 1∶3 and 1∶6 (REF:ORF57). A relatively small number of signals are affected by binding. REF-binding site of ORF57 is short and comprises residues 103–120.
(1.45 MB TIF)

Figure S6 NOE derived distance constraints used in the structure calculation of the REF^54–155^ and ICP27^103–138^ complex. The position of short and medium range NOE d-connectivities are shown in (A) for REF and (B) for ICP27, the protein sequence coloured blue highlights tags introduced in cloning. (C) The distribution of all NOEs on a per residue basis. White, light grey, dark grey and black shading of bars indicates the number of meaningful intra-residue, sequential (i+1), medium (2≤i≤4) and long (5≤i) range constraints. Two samples were used for structure determination of the ICP27^103–138^:REF^54–155^ complex, these contained one protein ^13^C/^15^N uniformly labelled at 1 mM plus the binding partner in unlabelled form at 2 mM. Small over-titration of the labelled component was necessary to observe the signals otherwise broadened in the equimolar complex. (D) Intermolecular NOE restrains used in structure calculations are shown schematically between the individual residues of REF and ICP27. Positions of two α-helices of REF are marked. Each line corresponds to a non-redundant NOE restraint. Dark green continuous lines represent NOEs obtained unambiguously from ^13^C edited, ^12^C-filtered NOESY-HSQC spectra. Additional NOEs represented by light green dashed lines were obtained from more sensitive standard 3D NOESY-HSQC spectra.
(1.15 MB TIF)

Figure S7 Example sections of 3D ^13^C edited, ^12^C-filtered NOESY-HSQC spectra showing intermolecular NOEs. Positive signals are coloured red and negative green. ICP27 ^1^H signal assignments are shown in blue (horizontal dashed lines and marks) and REF ^1^H assignments shown as vertical orange dashed lines. The experiment selects NOE cross peaks between ^1^H(^12^C) of unlabelled ICP27^103–138^ and ^1^H-^13^C moieties of ^13^C,^15^N-labelled REF^54–155^, therefore providing exclusively inter-molecular restraints.
(0.87 MB TIF)
