# Supplementary figures and images for: Structural Basis for the Recognition of Cellular mRNA Export Factor REF by Herpes Viral Proteins HSV-1 ICP27 and HVS ORF57
Source: PLoS Pathog. 2011 Jan 6;7(1):e1001244. doi: 10.1371/journal.ppat.1001244 (PMC3017119; doi:10.1371/journal.ppat.1001244)

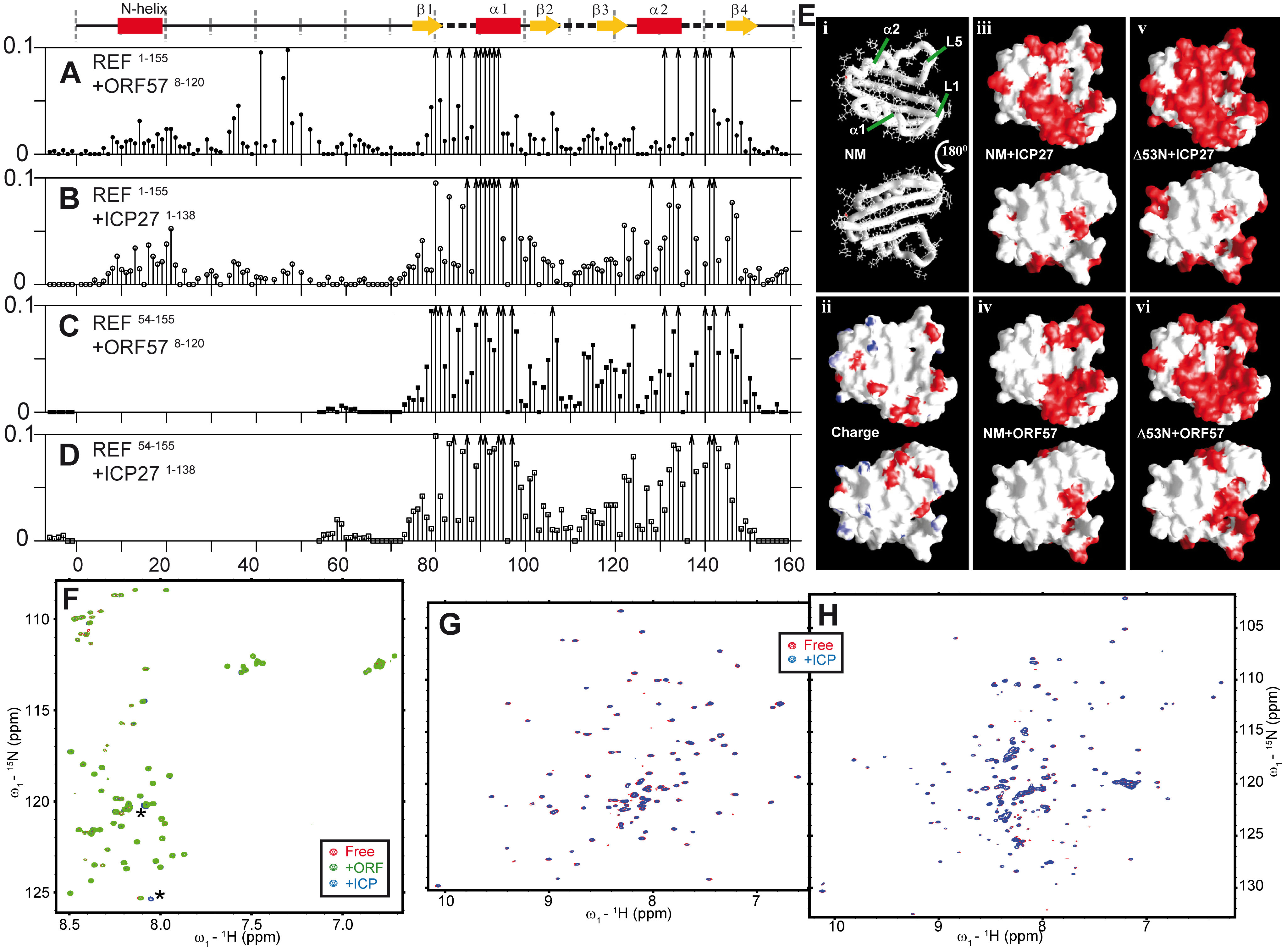

Supplement: Figure S1 — Overall identification of amino acid residues of REF2-I affected by binding with viral protein fragments plus SF2 and 9G8 spectra. Chemical shift changes within REF spectra were monitored upon addition of ICP27 or ORF57 constructs as an indication to which amino acids are involved in binding. Where the weighted chemical shift changes of amide signals δCS caused by complex formation were above 0.1, or the peak could not be followed due to broadening, an arrow is drawn. REF1–155 was titrated with ORF578–120 (A) and ICP271–138 (B), similarly REF54–155 was titrated with ORF578–120 (C) and ICP271–138 (D). Mapping of residues affected by binding is demonstrated (E). From the same titration data, ribbon representation of REF (i) is shown in the same orientation as surface representations showing charge (ii) with acidic and basic residues colored red and blue. Chemical shift changes from ORF57 (iv and vi) and ICP27 (iii and v) titrations are mapped with significant changes colored red. Labels used: NM, REF1–155; Δ53N, REF54–155. The similar pattern of shift changes throughout supports both ICP27 and ORF57 have the same main binging site situated in the folded RRM domain of REF. The changes to the chemical shifts within N-helix are likely to be caused by the release of N-helix which is normally bound to the same site in the free state of the protein [33]. (F) Overlay of 15N-HSQC spectra of the C-terminal region (residues 156–218) of REF2-I show no significantly changes from free form (red) upon addition of 2-fold excess of either ICP271–138 (blue) or ORF578–120 (green). Signals marked with asterisks originate from residues of C-terminal His-tag which are not part of REF. Additionally, 15N-HSQC spectra of the RRM domains of both 9G8 (G) and SF2 (H) show no changes upon addition of 2-fold excess of ICP27103–112 synthetic peptide. (3.88 MB TIF) [file ppat.1001244.s001.tif]

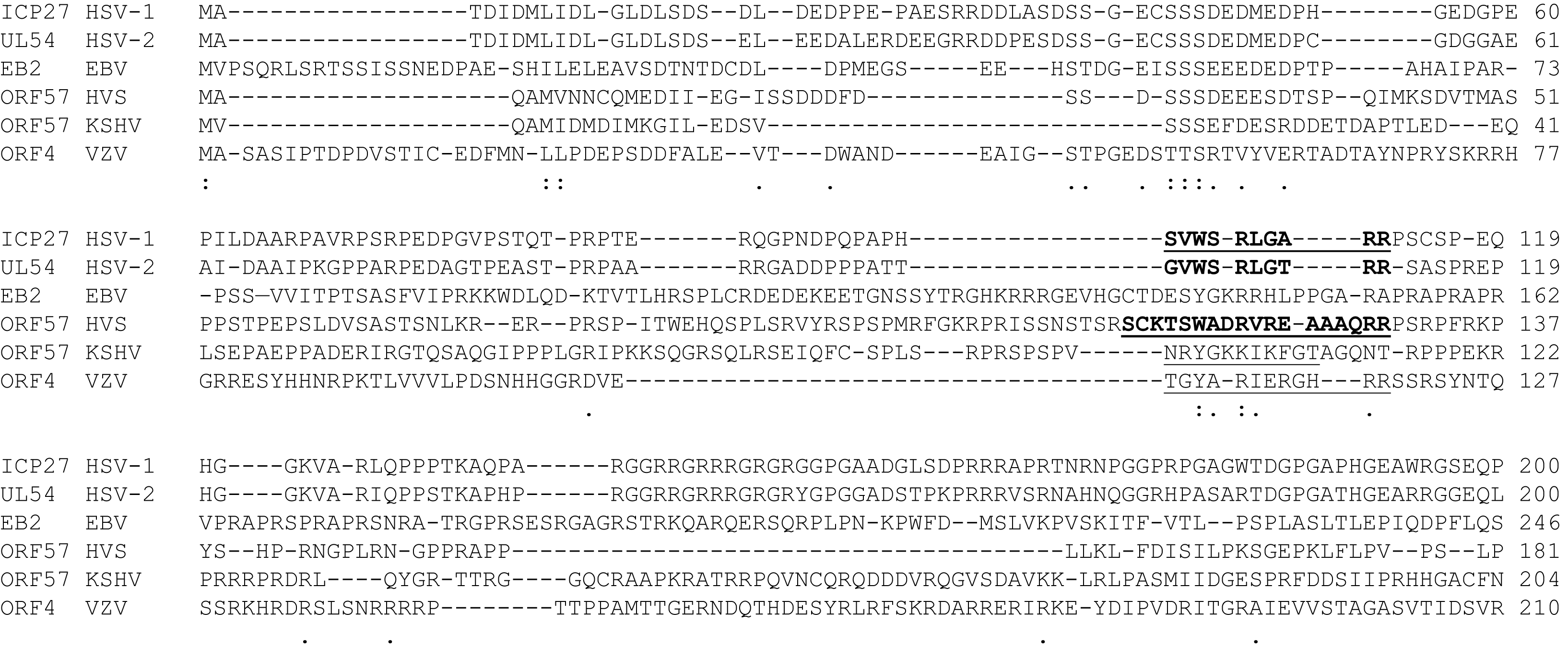

Supplement: Figure S2 — Sequence alignment of N-terminal parts of ICP27 homologues from α and γ herpesviruses. The first 200 amino acids of HSV-1 ICP27 (AAF43147) containing the REF-interaction site was aligned manually with the predicted unstructured N-terminal regions of the proteins HSV-2 UL54 (NP_044525), EBV EB2 (YP_401659), HVS ORF57 (AAA46125), KSHV ORF57 (YP_001129410) and VZV ORF4 (NP_040127). REF-interacting regions identified in this study are shown in bold, and regions probed for REF-binding using synthetic peptides are underlined. There is very weak homology between export adaptors within the N-terminal regions shown, with the exception of the very closely related HSV-1 ICP27 and HSV-2 UL54 proteins. (0.34 MB TIF) [file ppat.1001244.s002.tif]

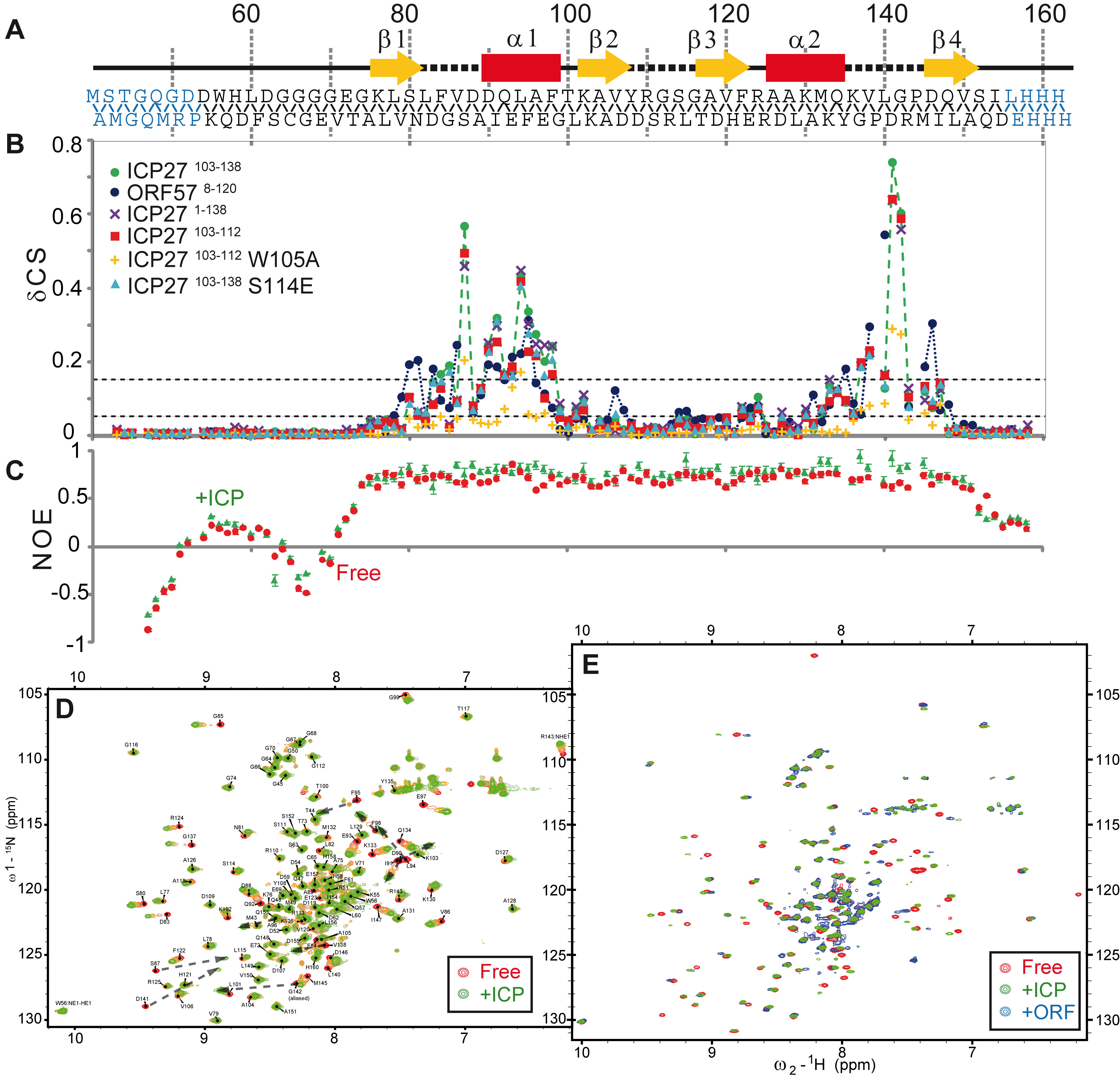

Supplement: Figure S3 — More detailed analysis of REF54–155 interactions with viral protein fragments and their mutants using NMR spectroscopy. (A) Amino acid sequence of REF construct (shown in a zigzag fashion), with T7 and poly-His tags coloured blue. Secondary structure elements are indicated with helices as red blocks, sheets as yellow arrows and larger loops as dotted lines. (B) Chemical shift changes δCS in backbone amides of REF54–155 upon addition of peptides: ICP27103–138, green circles & dashed line; ORF578–120, blue circles & dotted line; ICP271–138, purple diagonal crosses; ICP27103–112 synthetic peptide, red squares; ICP27103–112 W105A synthetic peptide, yellow plus signs; ICP27103–138 S114E synthetic peptide, green triangles. All the peptides were added in a 5-fold molar excess with respect to REF. (C) Heteronuclear 15N [1H] NOEs measured for REF54–155 in absence (red circles) and presence (green triangles) of ICP27103–138 were used as a measure of mobility change upon binding. (D) Overlay of 1H-15N-correlation HSQC spectra of REF54–155 with increasing amounts of ICP27103–138 added. Signal assignment is shown. Spectra are coloured red though green for free to bound forms of REF, respectively. Spectra are shown for the ratios 1∶0, 1∶0.5, 1∶1, 1∶1.5 and 1∶2 (REF:ICP27). (E) Comparison of TROSY spectra of REF54–155 in free form (red), bound to ICP27103–138 (green), bound to ORF578–120 (blue) suggests that the binding of ICP27 and ORF57 fragments affects essentially the same signals and hence occurs at the same binding site. All NMR experiments were carried out in the same NMR buffer (20 mM phosphate, 50 mM NaCl, 50 mM L-Arg/L-Glu/β-mercaptoethanol and 10 mM EDTA, pH 6.2 plus 10 mM DTT and 0.1% NaN3). (1.79 MB TIF) [file ppat.1001244.s003.tif]

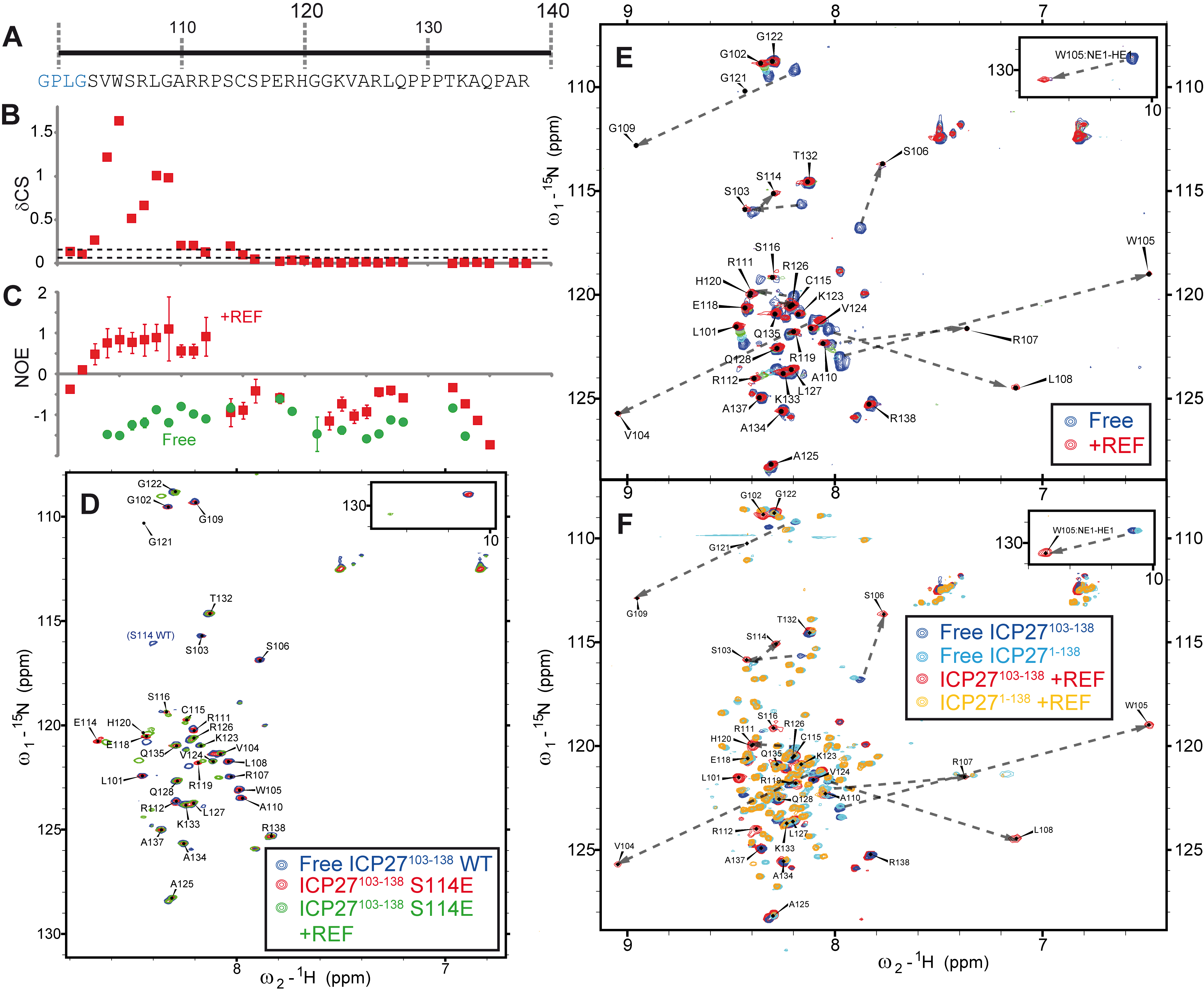

Supplement: Figure S4 — Analysis of ICP27103–138 interactions with REF54–155 using NMR spectroscopy. (A) Amino acid sequence of ICP27 construct. The sequence from the remaining PreScission protease cleavage site is coloured blue. (B) Chemical shift changes in backbone amides of ICP27103–138 upon addition of Ref54–155, red squares. The horizontal dashed lines represent thresholds for strong and medium shift changes used for creating the summary Fig. 2. (C) Heteronuclear 15N [1H] NOEs measured for ICP27103–138 in free form (green circles) and in presence of REF54–155 (red squares) were used to detect the change in polypeptide mobility. (D) Overlay of 1H-15N-correlation HSQC spectra of wild type ICP27103–138 (blue) with ICP27103–138S114E (red) in free form and ICP27103–138S114E with a 5-fold excess of REF54–155 added (green). The inset at the top right shows indole region of the spectrum. (E) Overlay of 1H-15N-correlation HSQC spectra of ICP27103–138 with various amounts of added REF54–155. Labels show sequence-specific signal assignment. Spectra are coloured blue (through green) to red for the free and complexed peptide respectively. For clarity only the 1∶0, 1∶0.25, 1∶0.5, 1∶0.75, 1∶2, and 1∶6 (ICP27:REF) spectra are shown. (F) Comparison of 15N-HSQC spectra of ICP27103–138 peptide in free (blue) and REF54–155 bound form (red) and ICP271–138 in free (cyan) and REF54–155 bound form (orange). Assignments for bound form of ICP27103–138 are shown. The spectrum of ICP27103–138 overlays well with the spectrum of ICP271–138, this suggests that the truncation does not disrupt the structure of the shorter construct. The REF-binding site of ICP27 1–138 is all situated within the 103–138 fragment, as signals from other parts of this longer viral protein construct are not affected by binding with REF. (1.42 MB TIF) [file ppat.1001244.s004.tif]

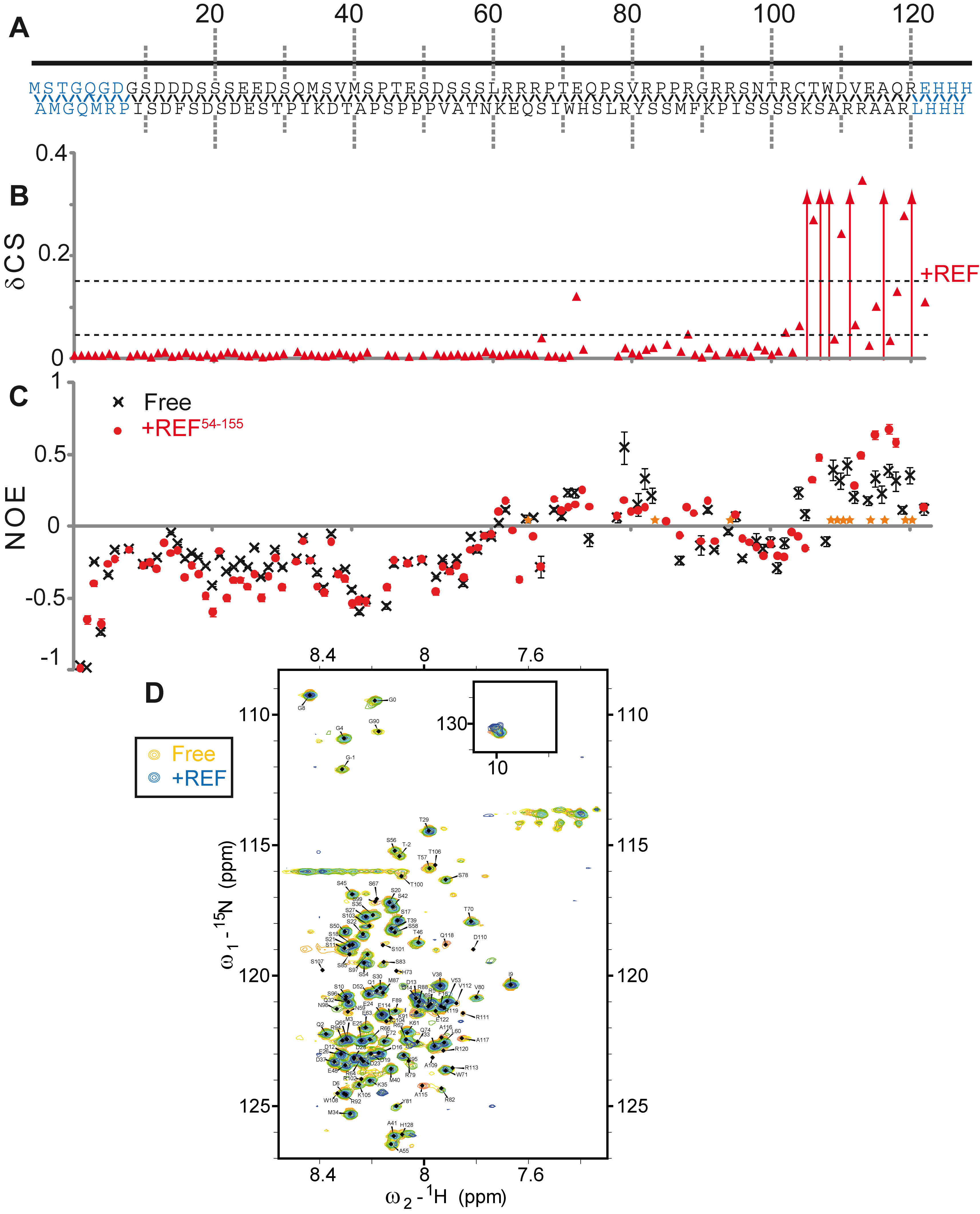

Supplement: Figure S5 — Analysis of ORF578–120 interactions with REF54–155 using NMR spectroscopy. (A) Amino acid sequence of ORF57 construct, with T7 and poly-His tags coloured blue. (B) Chemical shift changes in backbone amides of ORF578–120 upon addition of REF54–155 are shown as red triangles. The arrows identify residues broadened beyond detection in the bound state. The horizontal dashed lines represent thresholds for strong and medium shift changes used for creating the summary Fig. 2. (C) 15N [1H] NOEs measured for ORF578–120 in free form (black crosses) and in presence of REF54–155 (red squares) identify regions with polypeptide mobility changed upon binding. Orange stars mark residues with amide signals broadened beyond detection in the complex, for these no NOE data could be obtained. (D) Overlay of 15N-HSQC spectra for titration of ORF578–120 with REF54–155. Spectral assignment is shown. Spectra are coloured orange though blue for free to bound forms of REF respectively. Spectra are shown for the ratios 1∶0, 1∶0.15, 1∶0.3, 1∶1, 1∶3 and 1∶6 (REF:ORF57). A relatively small number of signals are affected by binding. REF-binding site of ORF57 is short and comprises residues 103–120. (1.45 MB TIF) [file ppat.1001244.s005.tif]

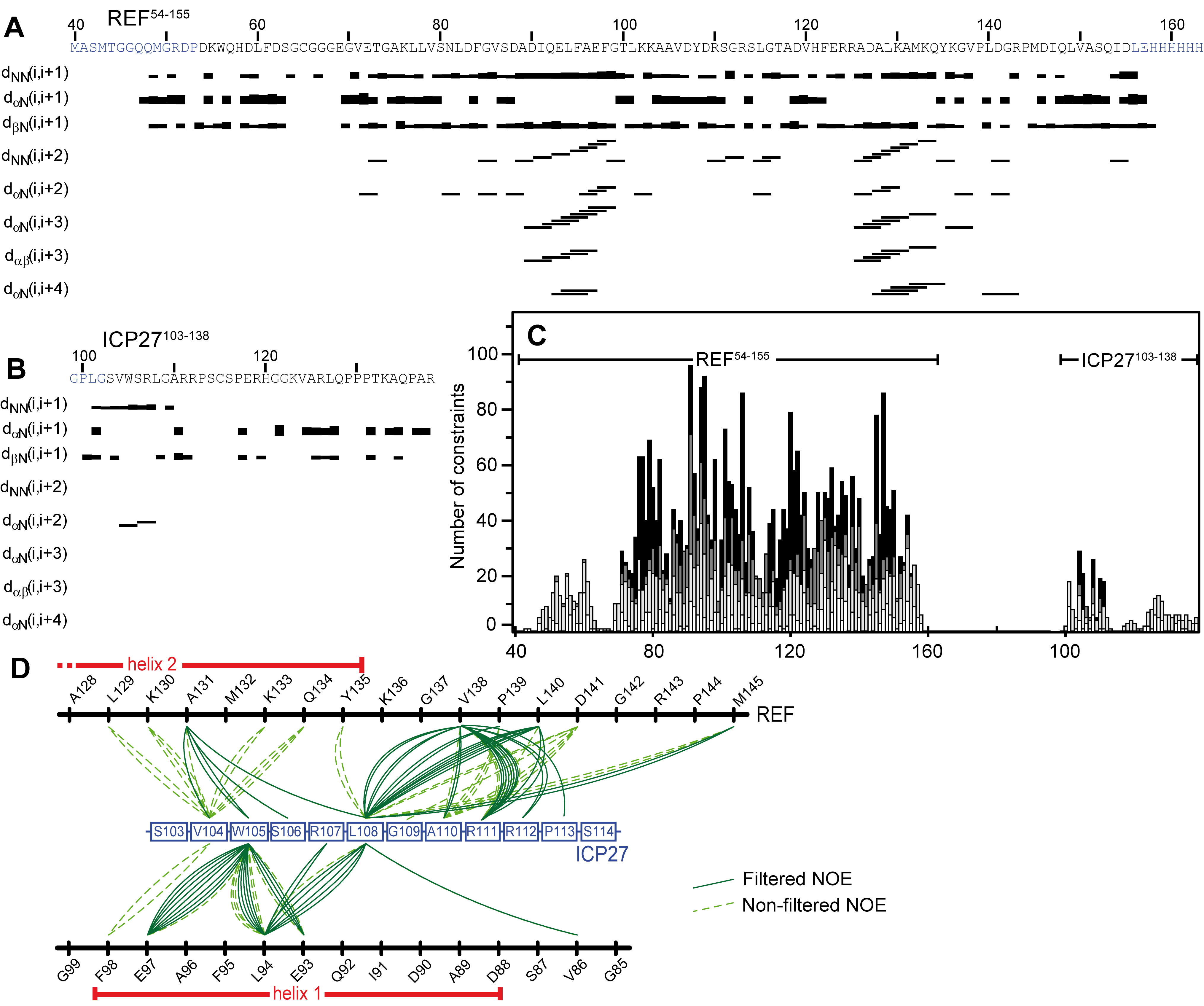

Supplement: Figure S6 — NOE derived distance constraints used in the structure calculation of the REF54–155 and ICP27103–138 complex. The position of short and medium range NOE d-connectivities are shown in (A) for REF and (B) for ICP27, the protein sequence coloured blue highlights tags introduced in cloning. (C) The distribution of all NOEs on a per residue basis. White, light grey, dark grey and black shading of bars indicates the number of meaningful intra-residue, sequential (i+1), medium (2≤i≤4) and long (5≤i) range constraints. Two samples were used for structure determination of the ICP27103–138:REF54–155 complex, these contained one protein 13C/15N uniformly labelled at 1 mM plus the binding partner in unlabelled form at 2 mM. Small over-titration of the labelled component was necessary to observe the signals otherwise broadened in the equimolar complex. (D) Intermolecular NOE restrains used in structure calculations are shown schematically between the individual residues of REF and ICP27. Positions of two α-helices of REF are marked. Each line corresponds to a non-redundant NOE restraint. Dark green continuous lines represent NOEs obtained unambiguously from 13C edited, 12C-filtered NOESY-HSQC spectra. Additional NOEs represented by light green dashed lines were obtained from more sensitive standard 3D NOESY-HSQC spectra. (1.15 MB TIF) [file ppat.1001244.s006.tif]

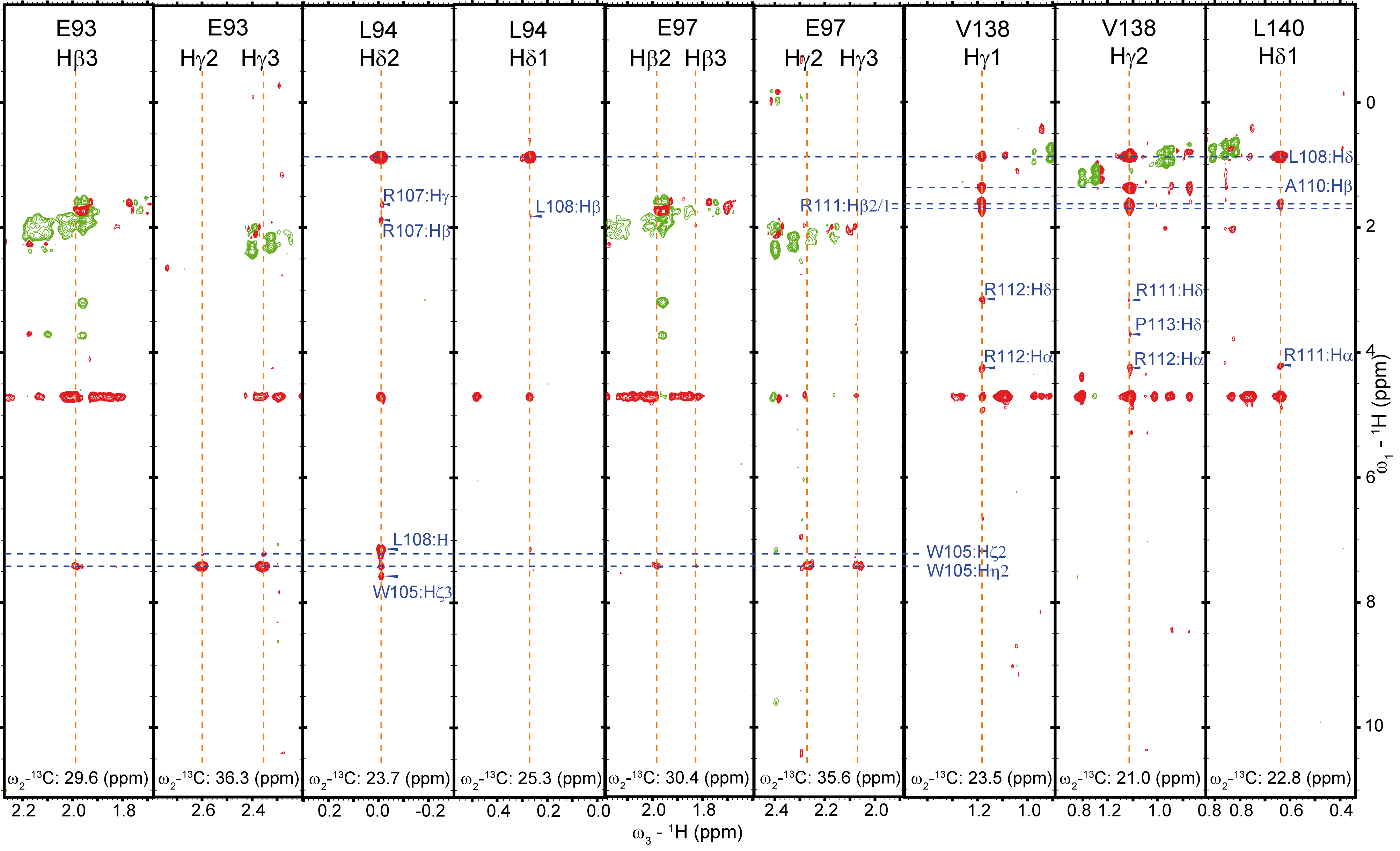

Supplement: Figure S7 — Example sections of 3D 13C edited, 12C-filtered NOESY-HSQC spectra showing intermolecular NOEs. Positive signals are coloured red and negative green. ICP27 1H signal assignments are shown in blue (horizontal dashed lines and marks) and REF 1H assignments shown as vertical orange dashed lines. The experiment selects NOE cross peaks between 1H(12C) of unlabelled ICP27103–138 and 1H-13C moieties of 13C,15N-labelled REF54–155, therefore providing exclusively inter-molecular restraints. (0.87 MB TIF) [file ppat.1001244.s007.tif]
